# Nrf1D Is the First Candidate Secretory Transcription Factor in the Blood Plasma, Its Precursor Existing as a Unique Redox-Sensitive Transmembrane CNC-bZIP Protein in Hemopoietic and Somatic Tissues

**DOI:** 10.3390/ijms19102940

**Published:** 2018-09-27

**Authors:** Jianxin Yuan, Hongxia Wang, Yuancai Xiang, Shaofan Hu, Shaojun Li, Meng Wang, Lu Qiu, Yiguo Zhang

**Affiliations:** Laboratory of Cell Biochemistry and Topogenetic Regulation, College of Bioengineering and Faculty of Sciences, Chongqing University, No. 174 Shazheng Street, Shapingba District, Chongqing 400044, China; 20161902043@cqu.edu.cn (J.Y.); 763116128whx@sina.com (H.W.); yuancaix@126.com (Y.X.); hufan2441@163.com (S.H.); 20161902030@cqu.edu.cn (S.L.); 20151901005@cqu.edu.cn (M.W.); qiulu99999@163.com (L.Q.)

**Keywords:** Nrf1, Nrf1D, secretory transcription factor, transmembrane, redox stress, alternative splicing, moving membrane-proteins, dynamic topobiology, unconventional protein secretion

## Abstract

Among multiple distinct isoforms, Nrf1D is synthesized from a de novo translation of an alternatively-spliced transcript of Nrf1 mRNA, as accompanied by a naturally-occurring deletion of its stop codon-flanking 1466 nucleotides. This molecular event leads to the generation of a reading frameshift mutation, which results in a constitutive substitution of the intact Nrf1’s C-terminal 72 amino acids (aa, covering the second half of the leucine zipper motif to C-terminal Neh3L domain) by an additional extended 80-aa stretch to generate a unique variant Nrf1D. The C-terminal extra 80-aa region of Nrf1D was herein identified to be folded into a redox-sensitive transmembrane domain, enabling it to be tightly integrated within the endoplasmic reticulum (ER) membranes. Notably, the salient feature of Nrf1D enables it to be distinguishable from prototypic Nrf1, such that Nrf1D is endowed with a lesser ability than wild-type Nrf1 to mediate target gene expression. Further evidence has also been presented revealing that both mRNA and protein levels of Nrf1D, together with other isoforms similar to those of Nrf1, were detected to varying extents in hemopoietic and somatic tissues. Surprisingly, we found the existence of Nrf1D-derived isoforms in blood plasma, implying that it is a candidate secretory transcription factor, albeit its precursor acts as an integral transmembrane-bound CNC-bZIP protein that entails dynamic topologies across membranes, before being unleashed from the ER to enter the blood.

## 1. Introduction

In the mid-1990s, nuclear factor-erythroid 2 (EF-E2) p45 subunit-related factor 1 (Nrf1, also called Nfe2L1, with accession NM_001130450 in GenBank, but it herein does not stand for the abbreviation of the nuclear respiratory factor) was identified as a key member of the cap’n’collar (CNC) basic-region leucine zipper (bZIP) family, which comprises Nrf2, Nrf3, Bach1, Bach2, Skn-1 and Cnc, in addition to p45 and Nrf1 [1,2,3,4]. Within this CNC-bZIP family, Nrf1 and Nrf2 are two principal master regulators of cognate target genes in mammals and are also widely expressed in various distinct tissues. Ever-increasing experimental evidence has demonstrated that Nrf1 exerts its unique functions, which are distinctive from Nrf2 [5]. This notion has been supported by the fact that Nrf1, but not Nrf2, is indispensable for maintaining cellular homoeostasis and organ integrity during normal development and growth, although both factors are required for diverse adaptive responses to a vast variety of intracellular and environmental stresses, which are also involved in most pathophysiological processes [6,7]. It is important to note that the essential function of Nrf1 is finely tuned by a steady-state balance between its production and concomitant processing through both post-transcriptional and post-translational pathways before being turned over. Notably, the processing of the *Nfe2l1* gene products ultimately leads to generating various lengths of mRNA transcripts and protein isoforms (with different and even opposing abilities) [8,9]. Overall, distinct Nrf1 isoforms are postulated together to confer cytoprotection on the host robust against cellular stress through coordinated regulation of distinct subsets of target genes. Transcriptional expression of these genes, particularly their basal expression, is predominantly driven by Nrf1 through binding to antioxidant response elements (AREs) or other homologous consensus sequences (i.e., AP-1 binding site) in those gene promoter regions.

In early studies using the consensus NF-E2/AP1-binding sites as a probe to clone the cDNA sequence of Nrf1, it was identified to consist of 742 aa in humans [1] or 741 aa in mice [10]. Similar cloning strategies were also employed to identify LCR-F1 [4] and TCF11 [2] that comprise 447 and 772 aa (with GenBank accession NO. U08853.1 and NM_003204.2), respectively. With the exception of length variations, both the nucleotide and amino acid sequences of LCR-F1 and TCF11 are fully identical with equivalents of Nrf1, and they are thus viewed as different length isoforms [5]. In fact, the prototypic Nrf1 (i.e., its full-length protein Nrf1α) is generated by translation from alternative splicing of mRNA to remove exon 4 that encodes ^242^VPSGEDQTALSLEECLRLLEATCPFGENAE^271^, called the Neh4L region, from human TCF11 [2]. Since Neh4L is lost in Nrf1, it was shown to exhibit similar transactivation activity to that of TCF11 [11], but this long TCF11 is not found in mice [10]. In addition, the post-synthetic processing of Nrf1/TCF11 may also yield multiple distinct polypeptides of between 140-kDa and 25-kDa, which together determine its overall activity to differentially regulate different target genes [8,9,12]. Further comparison of amino acid sequences demonstrates that LCR-F1 is a shorter form of Nrf1 (i.e., Nrf1β) [13], which is translated by its in-frame perfect Kozak initiation signal (5′-puCCATGG-3′) that exists around the methionine codons at between positions 289-297 in mice [1,2,10]. Thus, relative to Nrf1α, Nrf1β/LCR-F1 lacks the N-terminal acidic domain 1 (AD1) [11,14] and hence exhibits only a weak transactivation activity [4,8,15,16]. As such, Nrf1β/LCR-F1 activity may also be differentially induced in responses to distinct stressors [15,16,17]. In addition, Nrf1β/LCR-F1 is unstable because it may be rapidly processed to give rise to two small isoforms of 36-kDa Nrf1γ and 25-kDa Nrf1δ [8,9]; both may also be generated by additional in-frame translation. Among them, it is important to note that these two small dominant-negative Nrf1γ and Nrf1δ, when over-expressed, have a capability to competitively interfere with a functional assembly of the putative active CNC-bZIP transcription factors, so as to down-regulate expression of NF-E2/ AP1-like ARE-driven genes [8,15].

**Figure 1:**
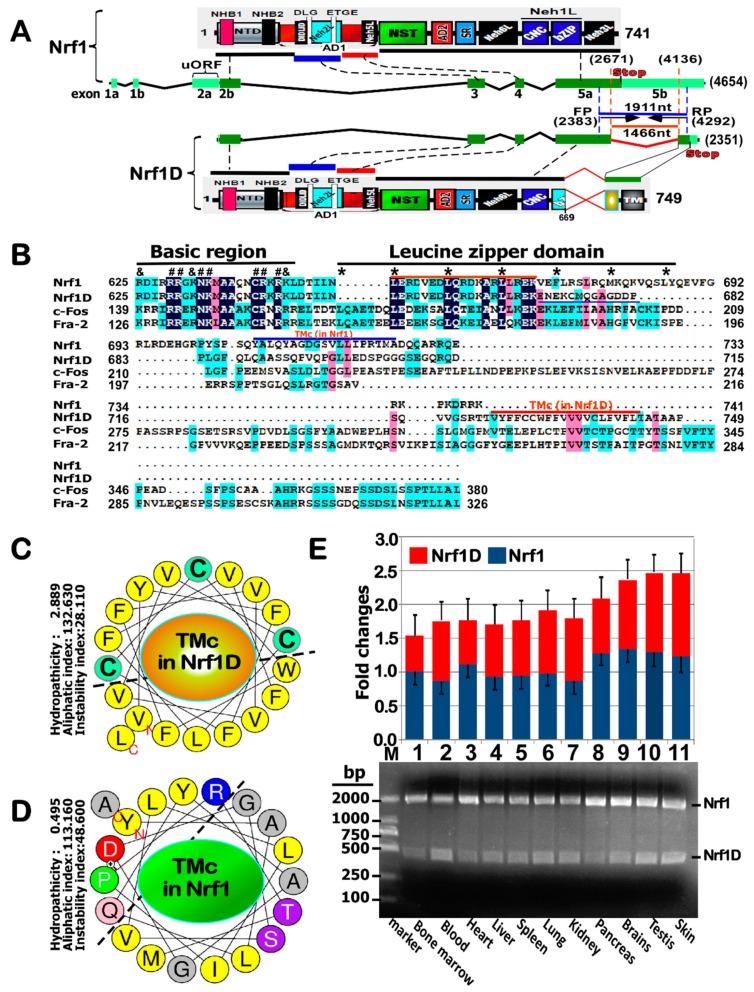
Structural differences in between Nrf1D and Nrf1 with distinct transcripts expressed in different tissues. (**A**) Schematic representation of structural domains of mouse Nrf1 and its splicing variant Nrf1D, which are translationally expressed by distinct exons of both transcripts, respectively. Both FP and RP stand for forward and reverse primers, respectively, besides other abbreviations described previously (see relevant references [5,9,12,18]) (**B**) Amino acid alignment of both bZIP and its C-terminal adjacent domains in Nrf1, Nrf1D, c-Fos and Fra-2. The basic region, leucine zipper domain, and TMc stretch are indicated. In the basic region, those residues critical for DNA binding to target genes are marked by symbols (#, &), while the stars (*) indicate the ‘*d*’ position of heptad repeats of leucine or other hydrophobic residues in the ZIP domain. (**C**,**D**) Two different α-helical wheels folded by those amino acids covering the C-terminal transmembrane (TMc) domains in Nrf1D and Nrf1. Within TMc of Nrf1D, the thiol-based Cysteine residues are placed in the green backgrounds, whilst hydrophobic and other aliphatic amino acid residues are in yellow backgrounds. (**E**) Differential expression of Nrf1 and Nrf1D transcripts in 11 different tissues from the mouse was determined by RT-PCR, with a pair of specific forward and reverse primers (i.e., FP and RP) described in the section of “Materials and Methods”. The data are here shown as a fold change (mean ± S.D.), each of which represents at least 3 independent experiments undertaken on separate occasions. In addition, the fidelity of both Nrf1 and Nrf1D transcripts was further confirmed by sequencing, as their nucleotide sequence alignment was shown in Figure S1.

Distinct other isoforms of Nrf1 have been determined to arise from multiple variants of mRNA transcripts, most of which are deposited in GenBank and Ensembl (i.e., ENSMUSG00000038615 and ENSG00000082641, representing mouse and human *Nef2l1* products, respectively). For example, those variants within the 3′- and 5′-untranslated regions were found to yield four different types of mRNA transcripts, that are hence consequently translated into distinct lengths of Nrf1 isoforms with different *trans*-acting potentials [2]. Additional isoforms were also identified to be generated from the alternative splicing of Nrf1 mRNA transcripts [15,16]. Among these variants, two that were originally designated as Nrf1 clones Δ767 and D are the most peculiar, with a deletion of its original translational start or stop codons, respectively. Removal of the first translation start codon results in the production of an N-terminally-truncated isoform designated as Nrf1^ΔN^ [12,13], within which the first N-terminal 181-aa region of Nrf1/TCF11 is replaced by an extra dodecylpeptide MGWESRLT- AASA. By striking contrast, the variant clone D of Nrf1 (with accession No. AF071084.1 in Genbank) does not only lack the intact original translation stop codon, but also acquires a substitutive change in the second half of its leucine zipper domain alongside with an extended C-terminal region, which is hence renamed Nrf1D (Figure 1A). However, both the biochemical characteristics and biological behaviors of Nrf1D have remained elusive to date.

To address the above issues on Nrf1D, we herein examine whether: (i) it has an unique C-terminal transmembrane (TMc) region, which is distinctive from the counterpart of intact Nrf1, involved in the topovectorial process; (ii) the TMc enables this variant to become more sensitive to oxidants and reducing agents, when compared with wild-type protein; and (iii) it is allowed for its putative processing to yield a putative specific isoform that is retained in the cytoplasm or secreted in the blood plasma, although most of this variant exists widely in somatic tissues.

## 2. Results

### 2.1. Nrf1D Has a Unique C-Terminal 80-aa Region with a Fold of Potential Redox-Sensitive TMc Entailed

By bioinformatic analysis of Nrf1D and Nrf1 (Figure 1A,B), it was revealed that the C-terminal 72-aa residues of wild-type Nrf1, which are extremely positively charged, are exchanged for an additional 80-aa stretch, which is, however, enriched with negatively charged residues in the variant Nrf1D. Further sequence alignment showed that the extra 80-aa region of Nrf1D, but not the equivalent sequence of Nrf1, appears to have a certain homology with c-Fos and Fos-related antigen-2 (Fra-2) (Figure 1B). Nonetheless, Nrf1D, rather than c-Fos or Fra-1, was also predicted to form a redox-sensitive (i.e., cystines at positions 729, 730 and 738) hydrophobic TMc α-helix folded on its C-terminal end (Figure 1C). The wheeled TMc region of Nrf1D has acquired an enhanced hydropathicity of 2.889, which is 5.8 times higher than the counterpart value (0.495) of Nrf1, despite of no marked difference in both aliphaticity (*cf. panels C with D*). Thus, it is postulated that Nrf1D is also tightly integrated through its C-terminal TMc region spanning across within the endoplasmic reticulum (ER) membranes, whereas the equivalent of Nrf1 could be loosely tethered to membranes with dynamic topologies entailed. In addition, the commonly-sharing basic-region and the first half of the leucine zipper domain of Nrf1D and Nrf1 are highly conserved with those of c-Fos and Fra-2. Collectively, it is therefore assumed that Nrf1D is better than Nrf1, acting as a substitute for c-Fos in the formation of a heterodimer with the latter partner c-Jun.

### 2.2. Nrf1D Exists Widely in Bloods, Bone Marrows and Somatic Tissues Examined

To investigate differences in the mRNA expression levels of Nrf1D and Nrf1 in distinct tissues, semi-quantitative real-time PCR was monitored by a pair of optimal primers that were complementary to their two commonly-sharing upstream and downstream nucleotide sequences across the putative alternatively spliced region, as indicated (Figure 1A). These results clearly demonstrated the presence of the variant *Nrf1D* in all tissues examined (Figure 1E). The expression profiles of *Nrf1D* mRNA were roughly similar to, and even higher than, the corresponding levels of wild-type *Nrf1*, particularly in mouse blood, lung, brains, testis and skin. All the shorter cDNA fragments of Nrf1D obtained from PCR products were further validated by cDNA sequencing, so that they were confirmed to be true by a multiple nucleotide sequence alignment (Figure S1).

**Figure 2:**
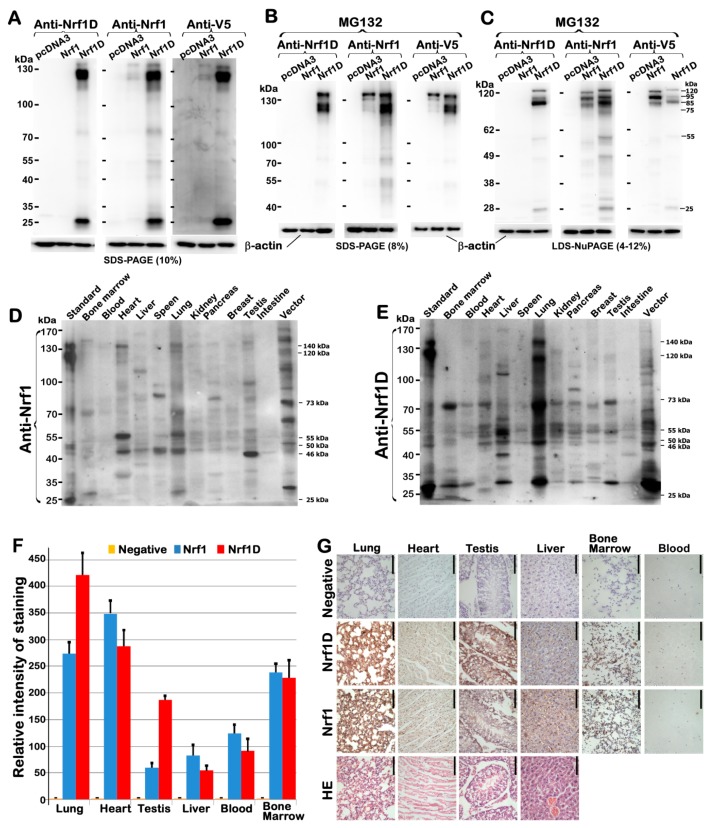
Differential expression of Nrf1D and Nrf1 proteins in mouse tissues examined. (**A**–**C**) Three independent experiments of COS-1 cells, that had been transfected with an expression construct for Nrf1, Nrf1D or empty pcDNA3.1 vector and then treated with the proteasomal inhibitor MG132 (5 μmol/L), showed that both Nrf1D and Nrf1 were identified by cross-immunoreactivity with three distinct antibodies against aa 192–741 in Nrf1, Nrf1D’s C-terminus-specific peptide, and their C-terminally-tagged V5 ectope, respectively. (**D**,**E**) Differential expression levels of Nrf1- and Nrf1D-derived proteins in different tissues from mice were examined by Western blotting with either Nrf1-recognized or Nrf1D-specific antibodies. (**F**,**G**) Anti-Nrf1D and -Nrf1 antibodies were also applied for immunohistochemical examinations of six different tissues, including lung, heart, testis, liver, bone marrow and blood (bar = 100 μm). Subsequently, the intensity of immunohisto- chemical staining in examined tissues was calculated and, is shown graphically (**F**). In addition, the first row “negative” images (**G**) were obtained from immunohistochemistry with the pre-immunized serum, instead of the primary antibody against Nrf1D-specific peptide, both of which was produced in the same animals.

To determine distinction in their protein expression profiles of between Nrf1D and Nrf1, its specifically-recognized antibody was made in our laboratory and then purified before being further verified by immunoblotting of total lysates of COS-1 cells that had been transfected with expression constructs for Nrf1D, Nrf1 or an empty pcDNA3 vector. The results showed that expression of Nrf1D was identified by its unique immunoreactivity specifically with the *per se* C-terminal peptide-specific antibody (Figure 2A, *left panel*), that was not cross-reactive with the N-terminal 670-aa region shared with Nrf1, implying this antibody is Nrf1D-specific.

Since Nrf1D and Nrf1 share a longer 670-aa portion, both were also recognized by additional two antibodies against aa 192–741 of Nrf1 (Figure 2A, *middle panel*) and its C-terminal V5 tag (Figure 2C, *right panel*), respectively. The mobility of Nrf1D polypeptides to electrophoretic locations of between 140-kDa and 25-kDa revealed that it appeared to have undergone the post-synthetic processing to give rise to its protein ladder. This event might occur probably through a similar mechanism accounting for Nrf1, as consistent with the notion reported previously [9,18]. Further immunobloting of Nrf1D and Nrf1, that had been allowed for ectopic expression of both proteins in COS-1 cells and then treated with 5 μmol/L MG132 before separation by two different electrophoresis systems (Figure 2B,C), revealed that Nrf1D appears to be more stable than Nrf1 protein. As such, Nrf1D also appeared to be subjected to the putative proteolytic processing in order to yield multiple distinct isoforms of between 120-kDa and 25-kDa, even in the presence of proteasomal inhibitor. Such difference in the proteolytic processing of between Nrf1D and Nrf1 may be attributed to the distinctive C-terminal regions within both CNC-bZIP proteins. This is also supported by additional evidence showing the remarkable existence of a stable 25-kDa polypeptide that has arisen from the putative processing of Nrf1D, but not Nrf1 (Figure 2A–C).

Further immunoblotting of different tissues unveiled that differential expression profiles of endogenous Nrf1D (Figure 2E) and Nrf1 (Figure 2D) were exhibited with distinct lengths of isoforms, even in each of the same organs. The variations in the protein expression of Nrf1D were detected in individual animals with gender differences (see the preprinted version of this paper that had been posted at bioRxiv 342428, https://doi.org/10.1101/342428). The full-length Nrf1D of ~140-kDa and its four major isoforms of ~115-, 70-, 45- and 35-kDa were determined primarily in the lung (Figure 2E). As such, some of Nrf1D and/or its derivates of between 115-kDa and 45-kDa were also, to greater or lesser extents, examined in other tissues, such as the blood, bone marrow, heart, liver, kidney, pancreas, brains and testis. By contrast, the wild-type Nrf1 of 140-kDa and its isoforms of between 90-kDa and 45-kDa were dominantly expressed in bone marrow, lung, heart and testis, apart from certain less abundances of them also detected in other tissues (Figure 2D). These different results demonstrate distributions of Nrf1D- derived isoforms are distinctive from that of multiple Nrf1 species in various tissues.

Further immunohistochemistry displayed distinctive staining with the Nrf1D-specific antibody, when compared with that obtained from anti-Nrf1 staining (Figure 2F,G, in which the first row “negative” images were obtained from the pre-immunized serum in the same animals that were employed to generate Nrf1D-specific antibody). This observation indicates differential expression of both CNC-bZIP proteins in the lung, heart, testis and liver, as well as in the bone marrow-derived and blood cells. However, it should also be noted that some immunochemical results are not completely consistent with the corresponding data obtained from Western blotting (as shown in Figure 2D,E). Such a subtle nuance suggests that an essential distinction between two experimental methods are likely determined by immunoreactivity with distinct conformations of antibodies- recognized polypeptides existing in the in situ or in-gel status, although relevant molecular details remain unknown.

**Figure 3:**
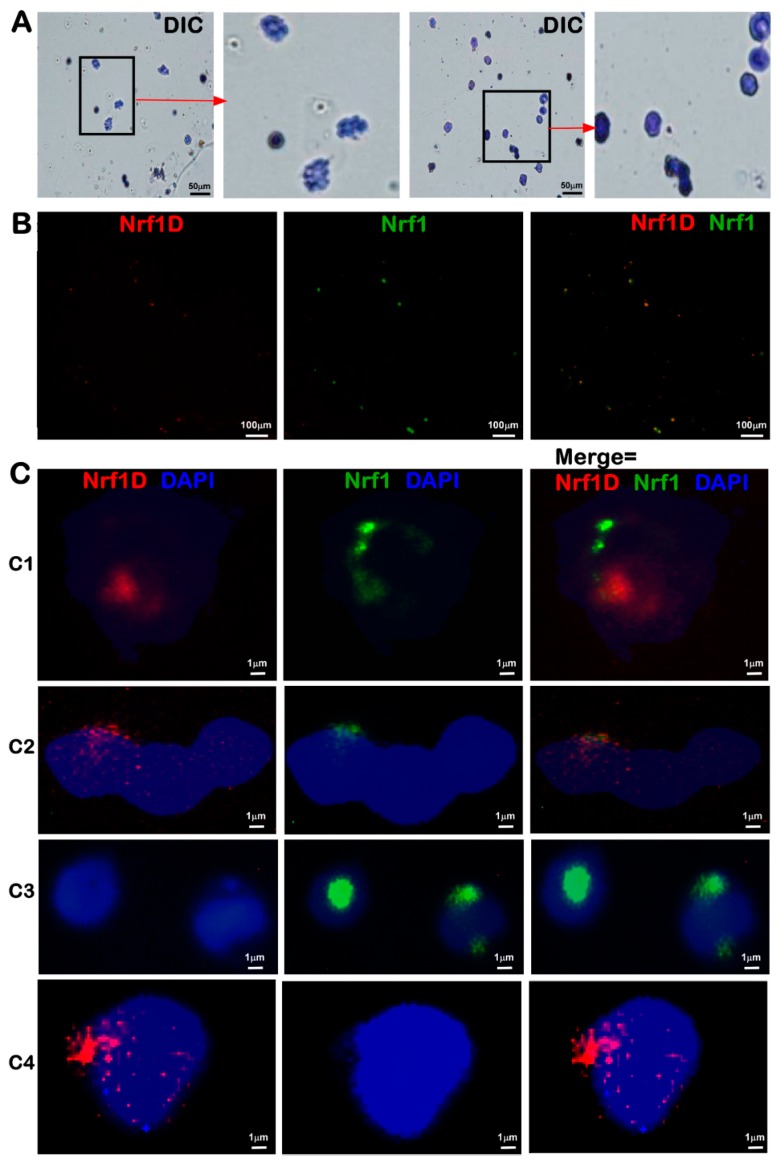
Fluorescence in situ hybridization (FISH) of both transcripts of Nrf1D and Nrf1 in bone marrow-derived cells. (**A**) Differential interference contrast (DIC) images (bar = 50 μm) of mouse bone marrow-derived cells were achieved in two different fields of vision by microscopy. (**B**,**C**) FISH of Nrf1D (red) and Nrf1 (green) transcripts expressed in bone marrow cells was conducted with their specific probes of Nrf1 (5′-CGCACGATGGCTGACC AGCAGGCTC-3′, which retained in Nrf1 but not Nrf1D, with its 5′-end labeled by 5-FAM, emitting a green fluorescence) and Nrf1D (5′-CGATTG CTTCGAGAAAAGGAAAATGAGAAGTGC-3′, which was labeled by Texas Red at its 5′-end so as to emit a red fluorescence). Nuclear DNA was stained by DAPI (blue). These images of indicated cells with different magnification (**B**, bar = 100 μm; **C**, bar = 1 μm) were acquired in different visual fields.

To clarify the presence of Nrf1D in the bone marrows, the fluorescence in situ hybridization (FISH) was also employed with a Nrf1D-specific probe (5′-CGATTGCTTCGAGAAAAGGAAAATG AGAAGTGC-3′, with a high fidelity to hybridize two small segments flanking its original mRNA alternatively-spliced and ligated sites). The resulting in situ hybridization of Nrf1D or Nrf1 mRNA transcripts by their cDNA probes was illustrated as red and green fluorescent signals, respectively (Figure 3B,C). The single signals of Nrf1D, Nrf1 and/or their superimposed signals, were displayed exclusively in the juxtanuclear locations of bone marrow-derived cells (in particular megakaryocytes and polykaryocytes), although this is required for further detailed studies.

### 2.3. Nrf1D Is a Candidate Secretory Transcription Factor

**Figure 4:**
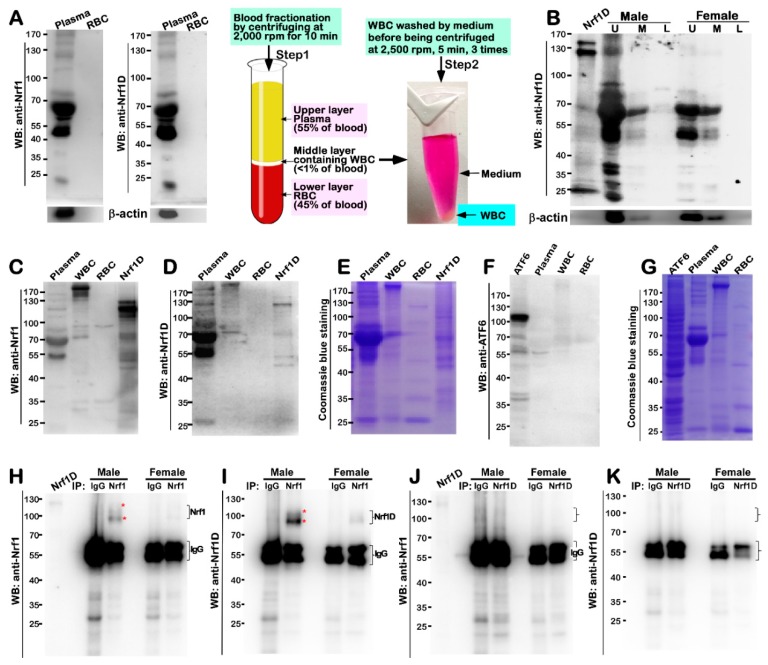
Detection of Nrf1D existing in mouse blood plasma. (**A**) Shows immunoblots of mouse blood plasma and RBC with anti-Nrf1 and Nrf1D-specific antibodies (*left upper two panels*). The procedure of blood fractionation by centrifuging at different speeds was illustrated (right two panels). (**B**) Distinct blood fractions from male and female mice were determined by Western blotting with Nrf1D-specific peptide antibody. The Nrf1D-expressed cell lysates served as a positive control. In addition, secreted β-actin was detected in blood plasma (*bottom*). Abbreviations: U, upper layer of plasma; M, middle layer containing WBCs; L, lower layer containing RBCs. (**C**–**G**) Further extracts from blood plasma, RBCs and WBCs were visualized by immunoblotting with Nrf1 antibody (**C**) or Nrf1D-specific peptide antibodies (**D**). But, similar blood fractions were not cross-immunoreactive with ATF6-specific antibodies (**F**). Total protein extracts from the blood fractions were also seen by Coomassie Blue staining (**E**,**G**). (**H**–**K**) Immunoprecipitation (IP) of the blood plasma from male and female mice (i.e., these samples were firstly clarified by pre-incubating with pre-immunized serum and Protein-G Sepharose beads) was employed with anti-Nrf1, anti-Nrf1D antibodies, or IgG (served as an internal negative control). Subsequent immunocomplexes were visualized by Western blotting with indicated antibodies. The star (*) indicates Nrf1D isoforms from immunoprecipitation (**H**,**I**).

Despite the existence of Nrf1D and Nrf1 in blood and hematopoietic cells, it is a curiosity that pushes us to examine whether both factors and/or their derivates come into emergence in the blood plasma. As shown here, the initial results unveiled an immunoreactivity of the plasma constituents (which also contained ~55-kDa IgG), rather than red blood cells (RBC), with antibodies against Nrf1 and Nrf1D (Figure 4A). Further immunoblotting with the Nrf1D-specfic antibody revealed a major ~120-kDa protein along with an obvious polypeptide ladder between 90-kDa and 25-kDa in the upper plasma (Figure 4B, *the second lane*), but a few of polypeptides between 70-kDa and 45-kDa were also detected in the middle, rather than lower, fractions of blood obtained from male mice. By sharp contrast, almost none of longer Nrf1D forms, except shorter polypeptides of between ~80-kDa and 45-kDa, were examined in those corresponding fractions of blood from female mice (Figure 4B, left three lanes). Nevertheless, it is of significant importance to note that nearly no isoforms of Nrf1D were detected in the lower fractions (Figure 4A,B), which were enriched with erythrocytes (45% of total blood), at the bottom of the centrifuge tube, although the wild-type Nrf1 and its homologues were *ab initio* isolated in erythroid hematopoietic cells [1,2,4].

The IgG-depleted plasma was further subjected to the immunoblotting with antibodies against Nrf1 (Figure 4C) and Nrf1D (Figure 4D). The results showed that Nrf1 and Nrf1D together with their derivates were migrated by electrophoresis to locations between 140-kDa and 45-kDa enriched in the plasma (*the left first lane*), when compared with the positive expression controls (i.e., *the lane labeled* by *Nrf1D*). However, very few longer Nrf1D proteins between 140-kDa and 100-kDa) were detected in the purified fractions of WBCs (i.e., leukocytes) and RBCs (i.e., erythrocytes), although with an exception that only a few of the minor polypeptides of between 90-kDa and 70-kDa were determined to have less immunoreactivity with antibodies against Nrf1 (Figure 4C), but not with Nrf1D-specfic peptide antibodies (Figure 4D). In addition, a possible non-specific immunoreactivity appeared to be ruled out by the parallel experiments, which demonstrated that none of additional transmembrane- bound ATF6 and processed polypeptides existed in the blood plasma (Figure 4F,G).

To further verify the existence of Nrf1 and/or Nrf1D in the blood plasma, their antibodies were utilized in distinct experimental settings of immunoprecipitation (IP, that was conducted after the experimental samples were clarified by pre-incubation with the pre-immunized serum from the same animals that were allowed for generating Nrf1D-specific antibodies). The resulting immuno- precipitates of between ~110-kDa and 95-kDa with Nrf1 antibody were not only recognized by this antibody *per se* (Figure 4H) and also visualized by Western blotting (WB) with another Nrf1D- specific antibody (Figure 4I). However, it is very regrettable that none of these precipitates were indeed obtained from Nrf1D-peptide antibody, because they were not detected by Western blotting with antibodies against Nrf1 (Figure 4J) or Nrf1D antibody itself (Figure 4K). Collectively, these results suggest a possibility that the antigen-specific peptide of Nrf1D is much likely to be buried in the interior of certain folding conformation, so that it cannot be exposed, hence leading to no access to cognate antibodies during immunoprecipitation. This notion appears to be supported by the finding that Nrf1D-expressing cell lysates served as a positive control, with a strong cross-reactivity with anti-Nrf1 antibody, but only a weak cross-reactivity was measured by Nrf1D-specific antibody (Figure 4C,D). Rather, the additional possibility cannot be ruled out that the putative processing of Nrf1D might also result in a proteolytic removal of its C-terminal peptides from this protein, such that none of the immunoprecipitates were captured. Moreover, the immunoprecipitates with Nrf1 antibody were pulled down principally from male mice, but less or none of them were observed in female mice (Figure 4H,I), implying a possible gender difference.

### 2.4. Dynamic Topovectorial Moving of Nrf1D in and out of the ER Is also Determined by Its Unique TMc Region

**Figure 5:**
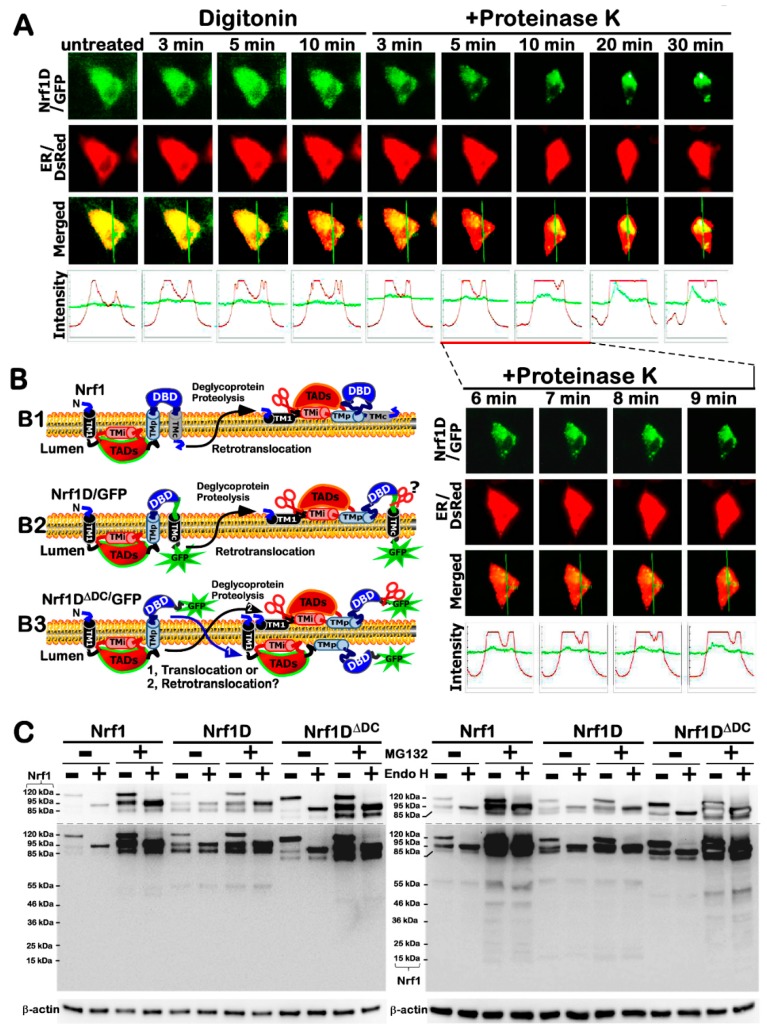
Dynamic repositioning of Nrf1D out of the ER membranes. (**A**) COS-1 cells that had been co-transfected with expression constructs for Nrf1D-eGFP and the ER/DsRed marker were subjected to live-cell imaging combined with in vivo membrane protease protection assay. The cells were permeabilized by digitonin (20 μg/mL) for 10 min, before being co-incubated with PK (50 μg/mL) for additional 30 min. During the course of the experiment, real-time images were acquired using the Leica DMI-6000 microscopy system. The merged images of Nrf1-eGFP with ER/DsRed are placed (*on the third row of panels*), whereas changes in the intensity of their signals are shown graphically (*bottom*). Overall, the images shown are a representative of at least 3 independent experiments undertaken on separate occasions. (**B**) Distinct membrane-topologies of Nrf1, Nrf1D-GFP, and Nrf1D^ΔDC^-GFP are dynamically moving in and out of the ER membranes, as depicted schematically. Transactivation domains (TADs), DNA-binding domain (DBD), and putative membrane-bound regions such as TM1, TMi, TMp and TMc are indicated. (**C**) Total lysates of COS-1 cells that had been transfected with expression constructs for Nrf1, Nrf1D or Nrf1D^ΔDC^ before being treated for 4 h with 5 µmol/L MG132, were subjected to in vitro deglycosylation reactions with Endo H (+) or without this enzyme (−) for additional 4 h at 37 °C. These reaction products were resolved by gradient LDS-NuPAGE gels containing 4–12% polyacrylamide and visualized by Western blotting with anti-V5 antibody.

Clearly, all those known secretory and transmembrane proteins, as elucidated in the current literature as far as we know [19,20], are biosynthesized by ribosomes budded with the ER. Therefore, we examined whether the membrane-topological folding of Nrf1D is also determined by its unique TMc region, which is distinctive from the equivalent of Nrf1 that is integrated within and around membranes [9,18,21] (Figure 5B1). To address this, the live-cell imaging of Nrf1D-eGFP (Figure 5A) and Nrf1D^ΔDC^-eGFP (lacking the unique C-terminal 80-aa region of Nrf1D, Figure S2A) combined with in vivo membrane protease protection assays, was performed to clarify whether these two fusion proteins have capability of being released from the ER into extra-ER compartments. COS-1 cells expressing Nrf1D-eGFP or Nrf1D^ΔDC^-eGFP, along with the ER/DsRed marker, were pre-treated for 10 min with digitonin (to permeabilize the cellular membranes) before being challenged with proteinase K (PK) for 3–30 min (still in the presence of digitonin) to digest cytoplasmic proteins. Hence, we surmised that if these two fusion proteins were transferred from the ER luminal side of the membrane into the cytoplasmic side and also dislocated into extra-ER compartments, it would become vulnerable to digestion by PK. As anticipated, the green fluorescent signals from Nrf1D- eGFP (Figure 5A) and Nrf1D^ΔDC^-eGFP (Figure S2A) were superimposed upon the red fluorescent images presented by ER/DsRed (an ER luminal-resident protein marker), implying that both fusion proteins are localized in the ER.

When compared to untreated cell status, almost no changes in the intensity of the green signals from Nrf1D-eGFP were observed within 10 min after the treatment of cells with digitonin and even in the presence of PK for 3 min (Figure 5A). This demonstrates convincingly that the GFP ectope attached to the C-terminus of Nrf1D was initially translocated into the ER and buried in the lumen of this organelle (Figure 5B2), so that it was protected by the membranes against PK digestion. As the time of additional PK treatment was further extended from 5 to 30 min, the green signals from Nrf1D-eGFP became gradually fainter, but did not disappear until the experiment was stopped (Figure 5A). By contrast, the ER/DsRed signals were not only reduced by PK digestion, but also obviously enhanced; this was attributable to the fact that the cells were shrunken in sizes during this treatment. Collectively, these observations indicate that a large fraction of Nrf1D-eGFP is dynamically repositioned from the ER lumen into extra-ER subcellular compartments (i.e., cyto/nucleoplasm), but another small fraction of this fusion protein is still tightly protected by ER membranes against PK digestion (Figure 5B2).

By comparison with Nrf1D-eGFP, it is much interesting to note that the green signals from Nrf1D^ΔDC^-eGFP were rapidly reduced following treatment of the cells with digitonin alone, and further diminished by additional PK digestion of this fusion protein for 3 to 10 min, albeit the time-lapsed signals were not eliminated by PK until the digestion time was extended to 30 min (Figure S2A). This observation, together with our previous work [9,18], indicates that a major fraction of Nrf1D^ΔDC^-eGFP (lacking the TMc region) was processed in close proximity to the ER, so as to be rapidly dislocated and released into extra-ER extracellular compartments, particularly after the cellular membranes was permeabilized by digitonin, but another small fraction of this mutant fusion protein was retained in the ER lumen and thus protected by the membranes (Figure 5B3). Collectively, the subtle nuance in the membrane-topological folding of between Nrf1D-eGFP and Nrf1D^ΔDC^-eGFP suggests that dynamic moving of Nrf1D in and out of the ER is also determined by its unique TMc region, beyond its N-terminal TM1 region as described in wild-type Nrf1.

### 2.5. The Post-Translational Processing of Nrf1D to Yield Distinct Isoforms in and out of the ER

In vitro endoglycosidase (Endo)-H reactions (Figure 5C) revealed that, like wild-type Nrf1 as described [9,18], Nrf1D was also *N*-glycosylated to become a ~120-kDa glycoprotein, which is then deglycosylated to yield another ~95-kDa deglycoprotein, prior to being proteolytically processed to generate an ~85-kDa N-terminally-truncated isoform. Together, the selective proteolytic processing of Nrf1D, as well as its C-terminally-truncated mutant Nrf1D^ΔDC^, is postulated to have occurred by a potential mechanism similar to that accounting for Nrf1, even in the presence of proteasomal inhibitor MG132 (Figure 5C and Figure S3).

The pulse-chase experiments showed subtle differences in the conversion of distinct isoforms arising from among Nrf1, Nrf1D and Nrf1D^ΔDC^ and their stability before being turned over (Figure S3). The half-lives of ~120-, 95- and 85-kDa isoforms yielded from Nrf1 were estimated to 0.33 h (=20 min), 1.40 h (=84 min) and 1.50 h (=90 min), respectively (Figure S3A, *right panel*), after Nrf1-expressing cells had been treated with 50 μg/mL cycloheximide (CHX, which inhibits biosynthesis of nascent proteins). Addition of MG132 caused a significant prolongation of the ~120-kDa Nrf1 glycoprotein’s half-life to 2.10 h (=126 min), implying that this proteasomal inhibitor may have an effect on conversion of Nrf1 into the 95- and 85-kDa isoforms. As such, the half-lives of the 95- and 85-kDa Nrf1 isoforms were also concomitantly extended to over 8 h, suggesting both protein degradation by proteasome-mediated pathways.

By contrast with Nrf1 isoforms, the abundance of ~120-kDa Nrf1D glycoprotein was much less than those levels of its 95-kDa deglycoprotein and processed 85-kDa proteins (Figure S3B, *left panel*), indicating that the ~120-kDa glycoprotein is rapidly converted into the 95-kDa deglycoprotein and processed 85-kDa isoforms. Such conversion of Nrf1D-derived isoforms and these protein stabilities were determined by distinctive extension of their half-lives to 0.88 h (=53 min), 2.46 h (=148 min) and 6.44 h (=386 min) respectively, after CHX treatment (Figure S3B, *right panel*), implying that Nrf1D-derived proteins are more stable than those equivalents of Nrf1. More intriguingly, further examinations revealed that the abundances of Nrf1D-derived ~120-, 95- and 85-kDa proteins were only marginally promoted by additional treatment of cells with MG132 (Figure S3B, *left panel*), with modest alterations of their half-lives estimated to be 0.63 h (=38 min), 3.13 h (=188 min) and 7.71 h (=463 min), respectively, after co-treatment with CHX (Figure S3B, *right graphs*).

The above finding indeed demonstrates that a considerable fraction of distinct Nrf1D-derived isoforms is irresponsive to the proteasome-mediated proteolysis. This fact suggests that they may also be targeted to a proteasome-independent degradation pathway, but it cannot be ruled out that a fraction of Nrf1D-derived isoforms may also be partially protected by its unique TMc-associated membranes against digestion by cytosolic proteases. To explore this possibility, the TMc-truncated Nrf1D^ΔDC^ mutant was also subjected to the pulse-chase experiments. This mutant protein was *N*-glycosylated into an ~110-kDa glycoprotein, and then further deglycosylated to yield a ~85-kDa deglycoprotein, before being proteolytically processed to give rise to an N-terminally-truncated ~75-kDa protein (Figure 5C and Figure S3C, *left panel*). The conversion of Nrf1D^ΔDC^-derived ~110-, 85- and 75-kDa proteins and their protein stability were also determined with distinct half-lives, that were estimated to be 0.69 h (=41 min), 0.53 h (=32 min), and 1.00 h (=60 min) respectively, after CHX treatment (Figure S3C, *right graphs*). Their half-lives were significantly prolonged by additional treatment with MG132 to 2.16 h or more than 8 h, respectively. Collectively, together with the data as described above (Figure S2A), these findings suggest that Nrf1D is tightly protected by its unique TMc-associated membranes, when compared with behavior of the TMc-truncated mutant Nrf1D^ΔDC^. Taken together with our previously-published results [9,12,18], it is therefore inferable that the ~110-kD glycoprotein of Nrf1D^ΔDC^ is rapidly repositioned into extra-ER compartments, whereupon it is allowed for deglycosylation digestion and then proteolytic processing of this mutant protein. The resulting ~85-kDa deglycoprotein of Nrf1D^ΔDC^ and its N-terminally-truncated ~75-kDa isoform have thus lost the putative protection by membranes, such that both proteoforms become more susceptible to protease attack, as compared to equivalents of the TMc-containing Nrf1D.

### 2.6. Nrf1D Has a Thiol-Based Redox-Sensitive Module in Its Unique TMc Region

**Figure 6:**
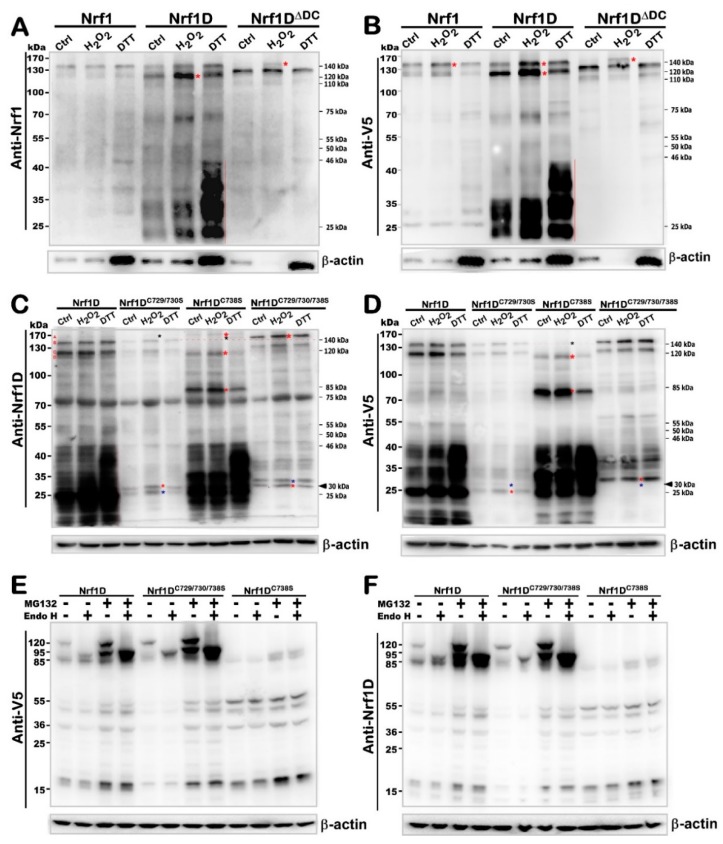
Redox reactions of Nrf1D and its mutants with H_2_O_2_ and DTT. (**A**,**B**) Total lysates of COS-1 cells that had been transfected with an expression construct for Nrf1, Nrf1D or Nrf1D^ΔDC^ were subjected to in vitro redox reactions with H_2_O_2_ (5 mmol/L) and DTT (5 mmol/L) for 10 min on ice, prior to being isolated by routine SDS-PAGE with two-layers made of 8% and 12% polyacrylamide, respectively and then examined by immunoblotting with antibodies against Nrf1 or V5 tag. (**C**,**D**) Similar redox Western blotting was also employed for further analysis of Nrf1D and its mutants (i.e., Nrf1D^C729/730S^, Nrf1D^C738S^ and Nrf1D^C729/730/738S^) that were incubated with H_2_O_2_ and DTT as described above. The redox reaction products were visualized by antibodies against Nrf1D or its C-terminal V5 tag. (**E**,**F**) COS-1 cells had been transfected with expression constructs for Nrf1D, Nrf1D^C738S^ and Nrf1D^C729/730/738S^ and treated with 5 μmol/L MG132 for 2 h before being harvested. The cell lysates were allowed for in vitro deglycosylation digestion by Endo H (+) or not (−). The resultant products were resolved by 4–12% LDS-NuPAGE gels and then determined by immunoblotting with indicated antibodies. Some specific bands were marked by stars (*, for detailed description, see the main text).

The above-described evidence demonstrates that the unique TMc region of Nrf1D dictates distinctions in the intrinsic regulation of this variant from wild-type Nrf1 and its mutant Nrf1D^ΔDC^. Together with bioinformatic analysis of distinct TMc regions in between Nrf1D and Nrf1 (Figure 1B–D), thereby, it is inferable that Nrf1D possesses a thiol-based (i.e., cystines 729, 730 and 738) redox-sensitive hydrophobic TMc module, that is topologically integrated within ER membranes (Figure 5B and Figure S2B). Here, to examine this thiol-sensitive module, total lysates of COS-1 cells that had been transfected with an expression construct for Nrf1, Nrf1D, or Nrf1D^ΔDC^ were subjected to in vitro redox reactions with 5 mmol/L of either hydrogen peroxide (H_2_O_2_) or dithiothreitol (DTT), respectively. These resulting reactants were analyzed by Western blotting with different antibodies against Nrf1 (Figure 6A) or its C-terminal V5 tag (Figure 6B). Of note, no obvious changes in the electrophoretic mobility of Nrf1 reactants with either H_2_O_2_ or DTT were examined herein. By sharp contrast, two longer Nrf1D isoforms of ~140- and 120-kDa (alongside with another ~75-kDa isoform) resolved on SDS-PAGE gels were obviously enhanced following oxidative reactions with H_2_O_2_, whilst reduction reactions with DTT led to significant increases in abundances of shorter molecular weight isoforms of between ~46-kDa and ~20-kDa (Figure 6A,B). However, conversely much less of these smaller isoforms were detected in Nrf1-expressing cell lysates. Collectively, these demonstrate that various lengths of TMc-adjoining polypeptides are progressively truncated from within Nrf1D, rather than Nrf1, particularly during reductive conditions. This notion is also further supported by the converse evidence that, indeed, almost none of similar shorter polypeptides were determined in Nrf1D^ΔDC^-expressing cell lysates, irrespective of whether they had reacted with H_2_O_2_ or DTT (Figure 6A,B). In addition, an extra polypeptide of Nrf1D^ΔDC^ with a heavier molecular mass of ~160-kDa was resolved by SDS-PAGE gels following its reactions with H_2_O_2_ but not DTT.

To determine whether the redox-sensitive TMc region of Nrf1D is thiol-site specific, three expression constructs for its mutants of triple, double or single cystines at positions 729, 730 and 738 into serine residues (i.e., Nrf1D^C729/730/738S^, Nrf1D^C729/730S^ and Nrf1D^C738S^) were made and then transfected into COS-1 cells, before redox reactions with H_2_O_2_ or DTT. Subsequently, Western blotting with Nrf1D-specific antibody revealed that almost all of other Nrf1D-derived isoforms between ~160-kDa and 25-kDa, except one ~75-kDa polypeptide, were substantially prevented by these two Cys-to-Ser mutants Nrf1D^C729/730S^ and Nrf1D^C729/730/738S^ (Figure 6C), but this seemed to be unaffected by redox reactions with H_2_O_2_ or DTT. Similar data were also obtained from anti-V5 antibody immunoblotting of Nrf1D^C729/730S^ and Nrf1D^C729/730/738S^ (Figure 6D). This indicates that these polypeptides arising from both Nrf1D^C729/730S^ and Nrf1D^C729/730/738S^ are unstable so as to be rapidly degraded, which results predominantly from Cys^729^/Cys^730^-to-Ser mutants. Further comparisons revealed that Nrf1D^C729/730/738S^, rather than Nrf1D^C729/730S^, conferred on two major Nrf1D-derived ~140- and ~120-kDa isoforms to be endowed with a slower electrophoretic shift to the locations of ~160- and ~130-kDa respectively (Figure 6C,D). Similar slower migration of small molecular weight isoforms between ~60-kDa and ~25-kDa was also examined in the case of Nrf1D^C729/730/738S^, but not of Nrf1D^C729/730S^ (Figure 6D). From this, it is inferable that a possible proteolytic processing event is blocked by the Cys^738^-to-Ser mutant in the contexts of Nrf1D^C729/730/738S^. Overall, different results from Nrf1D^C729/730S^ and Nrf1D^C729/730/738S^, when compared with Nrf1D, demonstrate that the membrane- topological folding of Nrf1D, its post-translational modification and/or proteolytic processing are influenced by its Cys-to-Ser mutants within TMc (leading to dual decreases in both its α-helical hydropathicity and site-specific instability, as calculated in Figure S2B).

The above assumption is also supported by further experimental evidence, revealing that the single Cys^738^-to-Ser mutant (i.e., Nrf1D^C738S^) caused almost all of Nrf1D-derived ~140- and ~120-kDa isoforms to be completely abolished and then replaced by additional two minor polypeptides of ~160-kDa and ~115-kDa (Figure 6C). The former ~160-kDa was not detected by immunoblotting with V5 antibody (Figure 6D), implying that its C-terminally-tagged V5 ectope may also be truncated. Surprisingly, changes of these longer isoforms were also accompanied by the emergence of an additional polypeptide of ~85-kDa arising from Nrf1D^C738S^, but not Nrf1D^C729/730S^ or Nrf1D^C729/730/738S^ (Figure 6C,D), suggesting that it is likely yielded from a potential proteolytic processing of unstable Nrf1D^C738S^-derived polypeptides. Interestingly, the existing longer isoforms of Nrf1D^C738S^ between ~160-kDa and ~85-kDa were further diminished by reactions with DTT, but not H_2_O_2_ (Figure 6C,D), indicating it become more unstable under the reductive conditions. By contrast, all those major polypeptides arising from Nrf1D^C729/730/738S^ and Nrf1D^C729/730S^ were modestly promoted by reactions with H_2_O_2_ and/or DTT (Figure 6C,D). Together with the aforementioned data (Figure 5, Figures S2 and S3), it is thus deduced that both Cys^729^ and Cys^730^ residues of Nrf1D are critical for TMc to serve as a thiol-based redox-sensitive module, whereas the adjacent Cys^738^ is indeed essential for monitoring the membrane-topological folding of TMc, its dynamic repositioning, and proteolytic processing to distinct isoforms. The conclusion is further supported by the converse evidence obtained from Nrf1D^C738S^; this mutant led to no obvious alterations in the abundances of smaller isoforms between ~46-kDa and ~20-kDa yielded from the mutant protein processing and their electrophoretic mobility, when compared with those arising from Nrf1D, which were distinctive from the equivalents of Nrf1D^C729/730/738S^ (Figure 6C,D). However, it was found that increased abundances of those lower molecular-weight polypeptides of between ~46-kDa and ~20-kDa arising from Nrf1D and Nrf1D^C738S^, but not Nrf1D^C729/730S^ or Nrf1D^C729/730/738S^, were examined following in vitro reactions with DTT rather than H_2_O_2_. Collectively, such reductive reactions enabled Nrf1D^C738S^ to become more vulnerable (than Nrf1D) to its selective proteolytic processing, implying requirements of the Cys^738^ residue for the proper folding of Nrf1D and its stability.

Next, to clarify whether the processing of Nrf1D and its TMc mutant proteins is affected by 24-h incubation of COS-1 and HepG2 cells to a redox stressor *tert*-butylhydroquinone (tBHQ) or DTT, these cells were examined by Western blotting with three different antibodies against Nrf1β, Nrf1D or its C-terminally-tagged V5. The results demonstrated that both Nrf1D^C729/730/738S^ and Nrf1D^C729/730S^ gave rise to two major isoforms between ~140-kDa and ~120-kDa with a slightly slower mobility than equivalents of Nrf1D (Figure S4A–C). Such two isoforms were, however, completely abolished by a single point mutant Nrf1D^C738S^, but appeared to be replaced by additional two isoforms of ~115-kDa and ~105-kDa. These two major proteins of Nrf1D^C738S^ were electrophoretically migrated to similar locations of the N-terminally-truncated isoforms arising from Nrf1D or Nrf1D^C729/730/738S^, and also unaffected by deglycosylaton digestion with Endo H (Figure 6E,F). Together with the above- described data (Figure 6C,D), these findings suggest that the Nrf1D^C738S^ mutant proteins are rapidly processed to remove different lengths of its N-terminal portions so as to yield several proteoforms of Nrf1D^C738S^ between ~115-kDa and ~14-kDa. These proteoforms arising Nrf1D^C738S^ and Nrf1D^C729/730/738S^, by comparison with those yielded from Nrf1D, were differently recognized by distinct antibodies against Nrf1β, Nrf1D or its C-terminally-tagged V5. Overall, these data indicate that the N-terminal and C-terminal portions of Nrf1D^C738S^ and Nrf1D^C729/730/738S^ are proteolytically processed in different ways. This notion is also further supported by additional evidence obtained from Western blotting of HepG2 cells that had been transfected with an expression construct for Nrf1D, Nrf1D^C729/730/738S^ or Nrf1D^C738S^. Furthermore, the electrophoretic mobility of most proteoforms was less or not influenced by treatment with tBHQ or DTT (Figure S4A–F). However, this was strikingly distinctive from the aforementioned in vitro results as shown (Figure 6C,D). The distinction could imply there exists an endogenous cytoprotective adaptation to redox stress.

### 2.7. Nrf1D Has Only a Low Ability to Transactive ARE-Driven Reporter Gene Induced by Redox Stress

**Figure 7:**
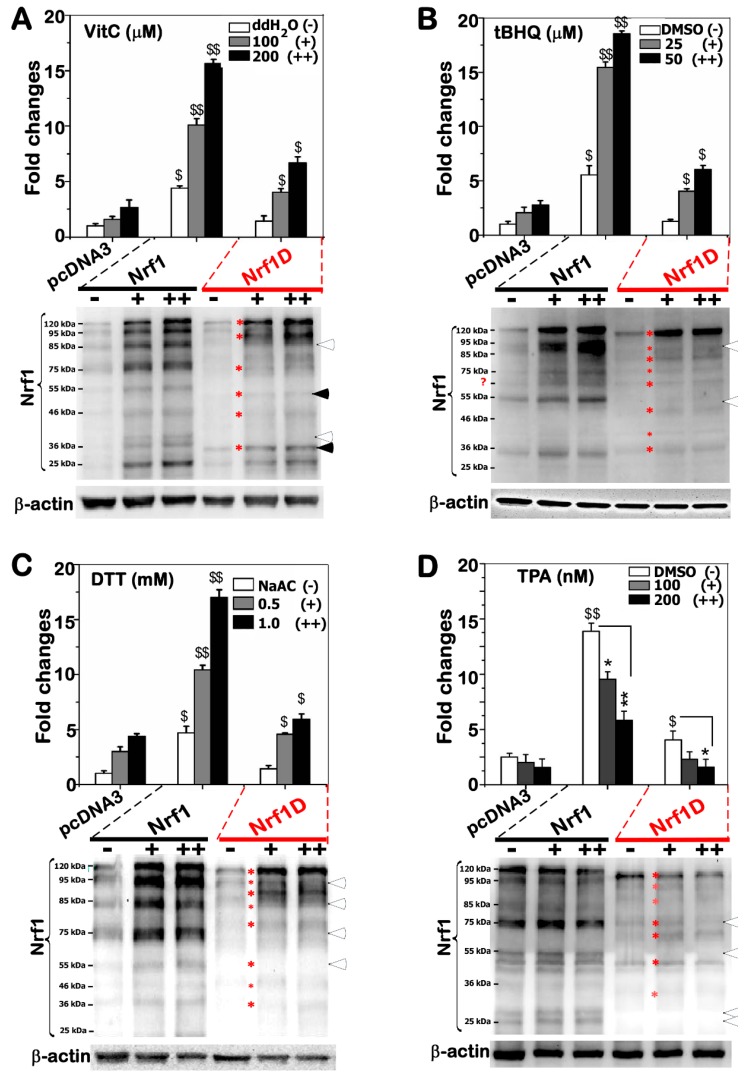
The activity of Nrf1D to regulate transcription of ARE-driven reporter gene in response to redox stress. COS-1 cells that had been transfected with an expression construct for Nrf1 or Nrf1D, together with *P_SV40_Nqo1-ARE-*Luc and pRL-TK reporters (at a ratio of 10:5:1 of their cDNAs) were treated with indicated doses of VitC (**A**), tBHQ (**B**), DTT (**C**) or TPA (**D**) for 24 h before being measured. Subsequently, basal and chemical-stimulated ARE-driven reporter activity regulated by Nrf1 or Nrf1D was calculated as a fold changes in its values against the background activity (i.e., measured following co-transfection of empty pcDNA3.1/V5 His B vector and ARE-driven reporter in response to indicated vehicle controls). Of note, the basal activity of empty vector was given the value of 1.0, and other data were calculated as fold changes (mean ± S.D.) relative to this value. The data presented each represent at least three independent experiments, each of which were performed in triplicate. Statistical analysis revealed significant increases ($ *p* < 0.05; $$ *p* < 0.01) and significant decreases (* *p* < 0.05; ** *p* < 0.01). Furthermore, these cell lysates were also resolved by 4–12% LDS- NuPAGE gels and then examined by Western blotting with V5 antibody. Relevant protein bands were indicated by stars (*).

To determine the Nrf1D activity to mediate ARE-driven reporter gene transcription, COS-1 cells were transfected with an expression construct for Nrf1D or Nrf1, together with *P_SV40_ nqo1-ARE-luc* and pRL-TK (as an internal control reporter), and then treated with distinct redox inducers. Figure 7A showed that Nrf1-mediated transactivation activity of ARE-Luc reporter gene was significantly induced to ~10–16-fold changes, as consistent with previous reports [22,23], by Vitamin C (i.e., ascorbic acid, which has dual redox ability to donate electrons enabling it to act as a free radical scavenger and also to reduce higher oxidation states of iron to Fe^2+^; the Fe reducing activity enables hydroxyl radical to be produced, hence exerting a pro-oxidant effect [24]). However, only ~4–7-fold changes in Nrf1D-mediated reporter gene transactivation by ascorbate were determined (Figure 7A), implying that Nrf1D has a lower activity than Nrf1 at mediating ARE-driven gene expression. Such a lower activity of Nrf1D could be attributable to less abundance of two processed polypeptides similar to ~85- and ~75-kDa arising from the mature processing of Nrf1 (Figure 7A, *lower panel*).

Further experiments revealed that Nrf1-mediated reporter gene was also substantially activated by stimulation of cells with either tBHQ or DTT, whilst Nrf1D only displayed a lower activity to transactivate the ARE-driven gene (Figure 7B,C). Intriguingly, treatment of cells with tBHQ or DTT caused a slightly faster electrophoretic migration of the full-length Nrf1D (of 749 aa) to ~110-kDa, only one longer polypeptide resolved by 4–12% LDS-NuPAGE gels, whereas intact Nrf1 (of 741-aa) was post-translationally processed to exhibit three or four major longer isoforms of between ~120-kDa and ~75-kDa (Figure 7B,C, *lower panels*). These findings indicate that the membrane- topological folding of Nrf1D and its proteolytic processing are certainly distinctive from those of Nrf1, particularly occurring under oxidative and/or reducing conditions. However, treatment of Nrf1-expressing cells with 12-*O*-tetradecanoylphorbol-13- acetate (TPA, a tumor promotor to induce AP-1 that comprises c-Jun and c-Fos) resulted in a significant decrease in the ARE-driven reporter activity (Figure 7D). Similar decreases in the basal and Nrf1D-mediated activity of the reporter gene were also determined. In addition, no marked changes in the abundances of Nrf1- or Nrf1D-derived isoforms were examined following TPA treatment, albeit they appeared to be unstable ((Figure 7D, *lower panels*). Taken together, Nrf1 (and/or Nrf1D) -mediated ARE-battery gene expression is likely repressed by TPA-stimulated transcription factors (i.e., AP-1).

### 2.8. Only a Smaller Fraction of Mature Processed Nrf1D Is Translocated in the Nucleus

**Figure 8:**
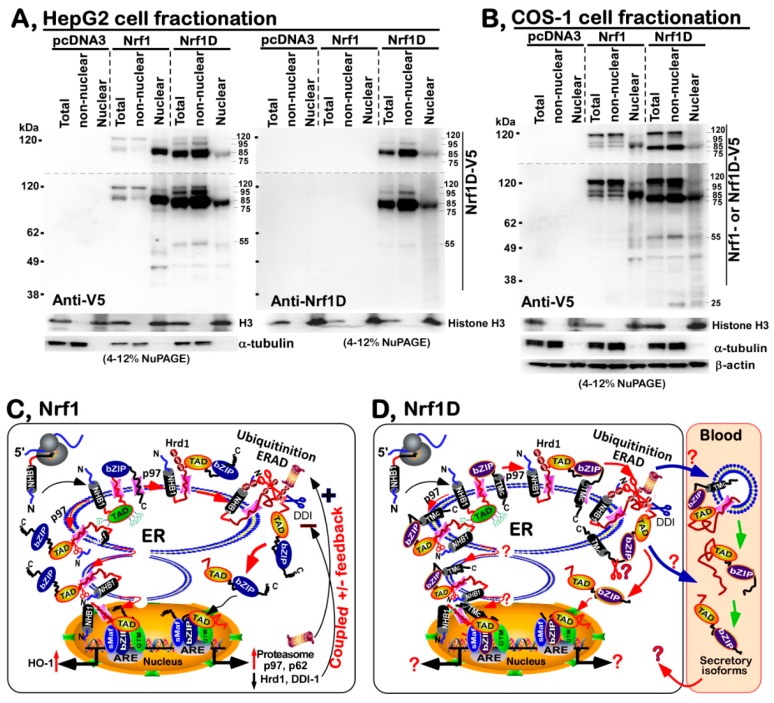
Two similar but different models proposed to explain distinctive topobiology of Nrf1D and Nrf1 in different subcellular distributions. Both lines of HepG2 (**A**) or COS-1 (**B**) cells expressing Nrf1 or Nrf1D were subjected to subcellular fractionation, followed by Western blotting of distinct subcellular fractions with different antibodies against Nrf1D or its C-terminal V5 tag, as well as other antibodies against the nuclear marker histone H3 or cytoplamic marker α-tubulin. The *upper panels* show the images obtained for exposure of the same immunoblots for a short time (**A**,**B**). The same samples were also visualized by immunoblotting with antibodies against Nrf1D (**A**, *right panel*) and its C-terminal V5 tag (**A**, *left upper two panels*). (**C**) A model is proposed to interpret dynamic membrane-topologies of Nrf1 folded within the ER and dislocated to the nucleus. Of note, distinct topovectorial processes of Nrf1 dictate its post-translational modifications and selective proteolytic processing to yield multiple isoforms, as well as its transcriptional activity to mediate target gene expression. In turn, the expression of Nrf1-target p97, DDI-1, and 26S proteasomal genes is also predominantly regulated by a processed mature Nrf1 factor through coupled positive and negative feedback circuits (this notion has been evidenced by our group). (**D**) Another similar but different model is introduced to explain topobiology of Nrf1D, that has a unique redox-sensitive TMc module spinning the ER membrane with an N_cyto_/C_lum_ orientation. Importantly, a few of Nrf1D isoforms are retained in the ER-associated cytoplasmic compartments or unlashed from the ER to enter the blood plasma (*right panel*), hence implying that it is the first candidate secretory transcription factor, albeit the detailed mechanisms remain to be further determined.

To explore the axiomatic reason why Nrf1D exerts a weaker activity than Nrf1 at transactivating the ARE-driven reporter gene expression as described above, the subcellular fractionation of HepG2 cells (Figure 8A) and COS-1 cells (Figure 8B) was employed to determine the nuclear localization of both CNC-bZIP proteins, together with their derivative isoforms. As expected, the results revealed that relatively smaller amounts of a major nuclear isoform of 85-kDa arised from the putative proteolytic processing of Nrf1D, by comparison with the equivalent arising from wild-type Nrf1, because this isoform was primarily recovered in the nuclear fractions of cells. In non-nuclear fractions, Nrf1 was principally represented by its full-length 120-kDa protein (Figure 8A), but surprisingly, Nrf1D was also dominantly recovered as minor 95-kDa and major 85-kDa isoforms in putative extra-nuclear locations. Of note, the massive abundance of the 85-kDa Nrf1D isoform was extra-nuclear, as compared to its nuclear amounts (Figure 8A, *right panel*). This difference in the subcellular fractions demonstrates that the full-length 120-kDa Nrf1 are processed to yield a major mature 85-kDa isoform and then translocated into the nucleus, whereas the full-length Nrf1D could be also rapidly processed (particularly while its anchored ER membranes were destructed during cell fractionation), in order to generate a minor 95-kDa and massive 85-kDa. Since only a smaller amount of 85-kDa Nrf1D was distributed to enter the nucleus, conversely the greater fraction of the non-nuclear 85-kDa proteins could be hence subjected to certain intracellular membrane-enclosed compartments and/or putative secretory pathways. In addition, small isoforms of Nrf1D, but not of Nrf1, between 55-kDa and 25-kDa C-terminally tagged by the V5 ectope were recovered in both the nuclear and non-nuclear fractions (Figure 8A,B). This implies that the unique C-terminus of Nrf1D enables an enhancement of its derived small protein stability.

## 3. Discussion

In the present study, Nrf1D is identified as a candidate secretory transcription factor, with its precursor existing as a unique redox-sensitive transmembrane-bound CNC-bZIP protein detected in several somatic tissues (e.g., heart, lung, liver and testis, as well as bone borrow), before being unleashed into the blood plasma. Collectively, a salient feature of Nrf1D is principally dictated by its C-terminal extra 80-aa region, allowing this variant protein to entail a similar, but different, membrane-topology from that of the prototypic Nrf1 (Figure 8C,D). In addition to the C-terminal distinction, it should also be noted that Nrf1D shares the longer N-termial 670-aa portion with Nrf1 (Figure 1). From this, it is inferred that both possess many common biochemical characteristics with some similar biological functions.

To give a better explicit interpretation of Nrf1 topobiology, an integral model (Figure 8C) is proposed on the basis of ever-accumulating evidence obtained from previous studies by both us [9,18,25,26,27] and other groups [28,29,30]. Upon translation of Nrf1, its newly-synthesized polypeptide is targeted and anchored within ER membranes through its NHB1 signal peptide, spinning in an N_cyto_/C_lum_ orientation (i.e., its N-terminal and C-terminal ends face the cytoplasmic and luminal sides, respectively). The NHB1-adjoining portions of Nrf1 are successively translocated into the ER lumen, in which it is *N*-glycosylated by oligosaccharyltransferases to yield an inactive glycoprotein, but its DNA-binding (CNC and bZIP) domains are retained on the cytoplasmic side of membranes (Figure 8C). When required for biological cues, portions of ER luminal-resident transactivation domain (TAD) elements will be dynamically repositioned through p97-fueled retrotranslocation [31] into extra-ER cyto/nucleoplasmic sides of membrane, whereupon Nrf1 is allowed for deglycosylation by *N*-glycosidases (e.g., NGLY1) and ubiquitination by Hrd1. Such modified Nrf1 protein is further subjected to the selective proteolytic processing to remove distinct lengths of its N-terminal portions by cytosolic DDI-1/2 protease-mediated cleavage and/or limited proteolysis by 26S proteasomes or calpains. In turn, transcriptional expression of p97, DDI-1, and proteasomal genes is predominantly regulated by processed mature Nrf1 factor through coupled positive and negative feedback circuits [26,27]. In addition, Nrf1 is also likely sorted out of the ER through putative membrane-ferrying to the outer nuclear membranes and then transported in the inner nuclear membrane to gain a direct access to target genes [25].

By contrast, Nrf1D is also a moving membrane-protein that entails dynamic topologies, which are much similar to, but slightly distinctive from, the folding of prototypic Nrf1 integrated within and around the ER membranes (Figure 8D). This distinction is determined by the unique C-terminal 80-aa region of Nrf1D, which only substitutes the C-terminal 72-aa residues of wild-type Nrf1 (Figure 1A). The C-terminal 80-aa region of Nrf1D is composed of a variant ZIP domain (that is certainly conserved with c-Fos and Fra-2, Figure 1B) and another integral membrane-spinning TMc stretch (Figure 1C). The variant bZIP of Nrf1D enables it to make distinctive dimerization from that made by the original bZIP of Nrf1, leading it to exert discrepant activity to mediate transcriptional expression of cognate target genes. However, the transcriptional activity of Nrf1D is tightly monitored by its distinct topovectorial processes, which are also determined by its unique redox- sensitive TMc module (Figure 8D), in addition to the NHB1 signal sequence, thus enabling it to be topologically folded, post-translationally modified and proteolytically processed in very similar, but different, fashions to account for the prototypic Nrf1 (Figure 8C).

In a combination with our previous studies of Nrf1 [9,18,25,26,27], the evidence that has been presented here reveals that Nrf1D is integrated in dynamic membrane-topologies within and around the ER (Figure 8D), which are also predominantly determined by both of its N-terminal signal anchor (i.e., NHB1) and its C-terminal redox-sensitive TMc module. Within Nrf1D, its amphipathic NHB1 peptide adapts an N_cyto_/C_lum_ orientation spinning cross ER membranes, whereas the relative hydrophobic TMc peptide is inferable to orientate in an N_cyto_/C_lum_ fashion across membranes. The acidic NST-containing TADs of Nrf1D are *cis*-translocated in the ER lumen, allowing it to be N-glycosylated, while its basic aa-enriched CNC and bZIP domains are retained on extra-luminal cyto/nucleoplasmic sides of membranes. Once some parts of the luminal TAD-adjoining portions of Nrf1D are dynamically repartitioned and repositioned by p97-driven retrotranslocation pathway from the ER lumen across membranes into extra-ER side [26,27,31], in which it should be allowed for deglycosylation digestion and further proteolytic processing by similar mechanisms accounting for Nrf1 (for the detailed descriptions, please see the references [26,27]). However, in a striking contrast with Nrf1, Nrf1D possesses its specific C-terminal redox-sensitive TMc module that enables this CNC-bZIP protein to exhibit its unique membrane-topological behavior. Thereby, this variant Nrf1D is also specifically processed in an additional way, that are distinctive from the Nrf1 processing. This is also supported by the data of live-cell imaging of Nrf1D (attached C-terminally to the GFP ectope) demonstrates that the local membrane-topology of TMc, though it has a higher hydrophobicity than the amphipathic NHB1 peptide, is still dynamically repositioned out of the ER, such that it is then dynamically dislocated into extra-ER cyto/nucleoplasmic compartments. Once Nrf1D enters these subcellular locations, it loses the membrane protection against digestion by cytosolic proteases (e.g., DDI-1/2, calpains and 26S proteasomes), in order to yield minor 95-kDa and major 85-kDa, along with several smaller isoforms of between 55-kDa and 25-kDa. This is also evidenced by subcellular fractionation revealing that among all Nrf1D isoforms, its full-length 120-kDa and 95-kDa isoforms were recovered in the non-nuclear, rather than nuclear, fractions, while other isoforms of between 85-kDa and 25-kDa were objectively present in both the nuclear and extra-nuclear fractions (Figure 8A,B).

Specifically, further experimental evidence unravels that the repositioning of Nrf1D and its selective proteolytic processing are finely tuned through a strict quality control by the precision thiol-based redox-sensitive module of TMc peptide. For example, the Cys^738^-to-Ser mutation of TMc enables it to give rise to an unstable protein of Nrf1D^C738S^, which could be allowed for fast dynamic repositioning into the extra-ER sides of membranes and rapidly proteolytic processing insomuch as that the putative glycoprotein and deglycoprotein of this mutant disappear abruptly and is replaced by several processed isoforms distinctive from equivalents arising from Nrf1D (Figure 6 and Figure S4). Furthermore, the di-cysteine-to-Ser mutant Nrf1D^C729/730S^ displays a relative slower electrophoretic mobility of glycoprotein and deglycoprotein, when compared with those of Nrf1D. This being the case, the redox-sensitive TMc of Nrf1D still enables this variant CNC-bZIP factor to be endowed with a less activity than wild-type Nrf1 to mediate ARE-driven reporter gene upon stimulation by redox stressors.

The low activity of Nrf1D is also attributable to a possible restriction of its juxtanuclear location detected in the bone marrow-derived cells (Figure 3). Importantly, further subcellular fractionation reveals that the low tranactivation activity of Nrf1D is much likely due to the fact that only a small fraction of the mature processed 85-kDa Nrf1D protein was translocated in the nucleus, but the majority of the 85-kDa fraction was still dominantly retained in other extra-nuclear compartments (Figure 8A,B). This finding therefore raises another possibility; much to our surprise, we discovered that some isoforms of Nrf1D are present in the blood plasma (Figure 4), although how it reaches the blood vessel is unclear. This exciting discovery demonstrates that Nrf1D has more than one possibly secretory isoform arising from the proteolytic processing of this variant CNC-bZIP transcription factor, which is released to enter the blood vessels (Figure 8D), albeit its precursor protein is existing as a unique transmembrane-bound way in hemopoietic and somatic tissues (Figure 2). However, the detailed topovectorial mechanisms whereby Nrf1D is proteolytically processed and then unleashed from membranes in order to be secreted into the blood plasma remains elusive. This is a major and difficult question to address where the intracellular Nrf1D is released and how it is delivered to the blood plasma. The plasma isoforms of Nrf1D may be derived from its founding mast cells and/or dendritic cells in the blood and other hematopoietic tissues [15], but the non-hematopoietic tissues cannot also be ruled out.

Apart from the classic secretory pathways, whether Nrf1D may also be postulated to act as an unconventional protein to be secreted into the blood plasma [32,33,34] should be taken into account. If Nrf1D is subjected to an unconventional protein secretory pathway, it will be unleashed from the ER membrane-tethered compartments and then enclosed within exosomes or other extracellular vesicles [35,36] before being released into the blood. This speculation also appears to be, at least in part, supported by the evidence that Nrf1D-derived isoforms of between 85-kDa and 25-kDa were stable in the nuclear and extra-nuclear fractions, whereas similar lengths of Nrf1-derived isoforms seemed to be rapidly degraded in the non-nuclear compartments, but this did not occur in the nucleus (Figure 8A,B). Such nuance indicates that Nrf1D isoforms of from 85-kDa to 25-kDa could be encompassed in a membrane-protected vesicles (e.g., exosomes) that will be secreted into the blood possibly by a not-as-yet identified pathway. As such, the existence of these vehicles involved in potential unconventional protein secretory pathways may also, indeed, partially support the idea of a transcription factor as Nrf1D released in the plasma to reach other peripheral tissues [32]. Once reaching target cells, the putative Nrf1D-containing vesicles will be internalized by fusion with the recipient plasma membranes so as to generate endosomes and then elicit its functionality in distinct target cells.

Notably, all the Nrf family proteins, such as NF-E2 p45, Nrf1 (with distinct lengths of isoforms Nrf1D, TCF11, LCR-F1/Nrf1β, and Nrf1^ΔN^) Nrf2 and Nrf3 were originally identified in the erythroid hematopoietic cells [1,2,3,4]. Importantly, the reticulocyte unravels a novel pathway of secretion during maturation into erythrocytes (reviewed in ref. [35]). Relevant multivesicular endosomes are found to associate with some small internal bodies, which are released after fusion of the endosomes with the plasma membrane of target cells. The novel form of putative secretion is the way by which the components of plasma membrane are discarded from maturing reticulocytes. Collectively, the exosome is defined for small membrane vesicles formed by vesiculation of intracellular endosomes and, thereafter, released by exocytosis [36]. However, it is unknown whether Nrf1D is secreted by exosomes during maturation and enucleation of erythrocytes and also internalized by endosomes in other recipient cells.

Moreover, it is crucial important to note that Nrf1D is highly expressed in the male testis rather than female ovary (Figure 2D–G and Figure S5, along with the preprinted version of this paper that had been posted at bioRxiv 342428, https://doi.org/10.1101/342428), and that the plasma existence of this variant CNC-bZIP transcription factor also implicates a possible gender difference, i.e., principally in male rather than female mice (Figure 4H,I). This finding thus raises another big open question of whether Nrf1D is secreted along with the release of the cytoplasmic contents during spermatogenesis.

## 4. Materials and Methods

### 4.1. Chemicals, Antibodies and Plasmids

All chemicals were of the highest quality commercially available. Among them, proteinase K (PK), Digitonin and Endo H were purchased from New England Biolabs, whereas MG132, CHX, DMSO, VitC, tBHQ, TPA and DTT were obtained from Sigma-Aldrich (St. Louis, MO, USA). Mouse monoclonal antibodies against the V5 epitope was from Invitrogen Ltd (Carlsbad, CA, USA). Of note, two specific antibodies against Nrf1 (aa 192–741) or Nrf1D-specific peptide (nearly at its C-terminus) were produced in rabbits by our laboratory in collaboration with commercial companies. Two expression constructs for Nrf1 and ER/DsRed had been described previously [14,25]. Here, Nrf1D was engineered by inserting the XbaI/SacII fragment (encoding aa 1–669 of Nrf1) with a Nrf1D-specific cDNA sequence (encoding its unique aa 670–749, which was synthesized by a commercial company) into the pcDNA3.1/V5-His B vector. Additional 3 cysteine-to-serine mutants Nrf1D^C729/730/738S^, Nrf1D^C729/730S^ and Nrf1D^C738S^ were created separately. The C-terminally-truncated Nrf1D^ΔDC^ was also made by only inserting the cDNA sequence encoding the N-terminal 669 aa of Nrf1. Furthermore, other expression constructs for two GFP-fusion proteins (i.e., Nrf1D-GFP and Nrf1D^ΔDC^-GFP) were generated by ligating their encoding sequences to the 3′-end of GFP-encoding cDNA into the KpnI/BstBI site of pcDNA3.1/V5-His B vector, respectively. The fidelity of all cDNA products was confirmed by sequencing.

### 4.2. Organ Collection and Blood Fractionation from Mice Approved in this Study

All of experimental organs and blood samples were collected from 8-weeks-old male or female BALB/c mice. After these mice were narcotized by diethyl ether, the blood samples were obtained from their eyeballs, that were perfused with PBS (phosphate buffer saline) containing 10 mU/mL heparin. The bloods were fractionated by centrifuging at 2000 rpm for 10 min for a collection of upper layer (i.e., blood plasma), middle layer containing white blood cells (WBCs), and lower layer containing red blood cells (RBCs). After WBCs and RBCs were rinsed by a serum-free medium, they were collected by centrifuging at 2500 rpm for 5min (which were repeated for 3 times), prior to being stored at −80 °C for the further use. Thereafter, the mice were sacrificed by being decapitated to obtain relevant tissues and organs, which were harvested and immediately snap-frozen in liquid nitrogen for further analyses. In addition, the bone marrow was also obtained from the tibiae and femurs of these mice by washing with PBS.

All mice used here were maintained under standard animal housing conditions with a 12-h dark cycle and allowed access *ad libitum* to sterilized water and diet. All relevant studies were carried out on 8-week-old male mice (with the license No. PIL60/13167 approved by Home Office on the 29th July 2011) in accordance with the United Kingdom Animal (Scientific Procedures) Act (1986) and the guidelines of Animal Care and Use Committees of Chongqing University and the Third Military Medical University, both of which were subjected to the local ethical review in China. Of note, collection of mouse organs and blood, along with other relevant experimental procedures were all conducted completely according to the valid ethical regulations that have been approved by the University Laboratory Animal Welfare and Ethics Committee (with the two institutional licenses SCXK-PLA-20120011 and SYXK-PLA-2017002).

### 4.3. Real-Time qPCR Analysis

Total RNAs were extracted from examined tissues and then reverse-transcripted to yield the first single-stranded cDNAs, which served as temples of real-time quantitative PCR, according to the manufacturer’s protocol (TaKaRa, Dalian, China). To investigate the differential expression of both Nrf1D (Genbank No. AF071084.1) and Nrf1 (Genbank No. NM_008686.3) at their endogenous mRNA levels, a pair of optimal primers complementary to the indicated upstream (5′-GCTGACTTC CTGGACAAGCAGATGA-3′) and downstream (5′-GGAACCAACCACCTGGCTATC-3′) nucleotide sequences were designed and then added in the PCR. The reaction was conducted in the following conditions: Inactivation at 94 °C for 2 min followed by 34 cycles of 10 s at 98 °C denatured, 30 s at 58 °C annealed, and 2 min at 58 °C extended, with an additional extension of 7 min before being stopped. The resulting PCR products were isolated by 1% agarose gel electrophoresis and collected for cDNA sequencing. In addition, expression of β-actin or RPL13A mRNAs was used as an internal control for normalization.

### 4.4. Fluorescence In Situ Hybridization (FISH)

The bone marrow cells were fixed in 10 mL of 4% paraformaldehyde for 15 min at 4 °C, and pelleted by centrifuging at 1000 rpm for 5 min. After the supernatants were abandoned, the cells were permeabilized for 10 min by 0.1% Triton X-100 at normal temperature, and then collected by centrifuging at 1000 rpm for 5min. The cells were adjusted to an appropriate concentration of 3 × 10^6^/mL of PBS and dropped on glass slides. Subsequently, the slides were immersed in a 2× SSC solution at 37 °C for 5 min, placed in a proper order of 70%, 85% and 100% ethanol at room temperature for 3 min each for dehydration, and then dried naturally. A hybridization solution was preheated at 58 °C for 2 h, and then added to the above slides at a total volume of 10 μL, which contained 1 μL of each probe of Nrf1 (5′-CGCACGATGGCTGACCAGCAGGCTC-3′, which retained within Nrf1 but lacked in Nrf1D, with its 5′-end labeled by 5-FAM, emitting a green fluorescence) and Nrf1D (5′-CGATTGCTTCGAGAAAAGGAAAATGAGAAGTGC-3′, which was labeled by Texas Red at its 5′-end so as to emit a red fluorescence). These probes were allowed to hybridize with indicated samples overnight at 37 °C in a hybridization chamber. The probe-hybridized sample slides were washed in 0.4× SSC plus 0.3% Tween-20 at 65 °C for 3 min, followed by 2× SSC plus 0.1% Tween-20 at room temperature for 30 min, and then stained with Wright-Giemsa and DAPI, respectively. All probes used in this study were synthesized by Invitrogen (Shanghai, China).

### 4.5. Cell Culture, Transfection, and Reporter Gene Assays

Unless otherwise indicated, monkey kidney COS-1 cells (3 × 10^5^, which had been previously purchased from ATCC and maintained in our laboratory) were seeded in 6-well plates and grown for 24 h in Dulbecco’s Modified Eagle Medium (DMEM) containing 10% fetal bovine serum (FBS). After reaching the 70% confluence, the cells were transfected with a Lipofectamine 3000 (Invitrogen) mixture that contained an expression construct for Nrf1, Nrf1D or mutants, together with two reporters *P_SV40_Nqo1-ARE*-Luc and pRL-TK (at a ratio of 10:5:1 of their cDNAs). The latter reporter was used as an internal control for transfection efficiency, whilst an additional mutant version of these reporter genes that lacked ARE sequences served as a negative control. At 24 h after transfection, the cells were further treated by different doses of DTT, tBHQ, VitC or TPA for an additional 24 h. Subsequently, ARE-driven luciferase reporter activity and western blotting were measured approximately 24 h after transfection alone or plus chemical treatments. Basal and stimulated reporter gene activity regulated by Nrf1, Nrf1D or mutants was calculated as a ratio of its values against the background activity (i.e., measured following co-transfection of empty pcDNA3.1/V5 His B vector and ARE-driven reporter in cellular response to indicated vehicle controls). Of note, the basal activity of empty vector was given the value of 1.0, and other data were calculated as fold changes (mean ± S.D.) relative to this value. The data presented each represent at least three independent experiments, each of which were performed in triplicate, followed by statistical analysis of significant differences.

### 4.6. Live-Cell Imaging Combined with In Vivo Membrane Protease Protection Assays

The live-cell imaging was performed as described by [8,9]. COS-1 cells (2 × 10^5^) were seeded in 35-mm dishes and cultured overnight. The cells were then co-transfected for 6 h with 3 μg DNA of an expression construct for Nrf1D-GFP or Nrf1D^ΔDC^-GFP and 0.5 μg DNA encoding ER/DsRed (as an ER luminal-resident protein marker [14,18]). Subsequently, the cells were allowed for a 16-h recovery from transfection, the plasma membranes of COS-1 cells were permeabilized by digitonin (20 μg/mL) for 10 min. Thereafter, the cells were subjected to in vivo membrane protection reactions against digestion by PK (50 μg/mL) for 35 min. During the experiment, live-cell images were acquired every minute on Leica DMI 6000 green and red fluorescence microscopes equipped with a high-sensitivity HAMAMATSH ORCAER camera. Relative fluorescence units were measured by using the Simulator SP5 Multi-Detection system for GFP with 488-nm excitation and 507-nm emission, and for DsRed2 with 570-nm excitation and 650-nm emission.

### 4.7. Deglycosylation Assay, Western Blotting and Coomassie Brilliant Blue Staining

Experimental cell lysates are subjected to in vitro deglycosylation digestion by Endo H, according to the manufacturer’s instruction (New England Biolabs, Ipswich, MA, USA). Equal amounts of proteins prepared from cell lysates were loaded into each of electrophoretic wells. They were subjected to protein separation by two distinct electrophoresis systems: SDS-PAGE and LDS-NuPAGE gels as described previously [5,18], followed by visualization by Western blotting with distinct antibodies against Nrf1, Nrf1D or its C-terminally-tagged V5 ectope. β-Actin served as an internal control to verify amounts of proteins loaded in each well. On some occasions, nitrocellulose membranes that had already been blotted with antibodies were rinsed for 30 min with a stripping buffer before being re-probed with additional primary antibody [37]. Furthermore, some protein-blotted gels or nitrocellulose membranes were stained by 2.8 μg/mL of Coomassie brilliant Blue R-250 in 10% acetic acid. After the protein bands appeared in 30 min, the gels were de-stained in 10% acetic acid for over 2 h.

### 4.8. Immunoprecipitation and Immunohistochemistry

Blood samples were extracted in RIPA lysis buffer (Beyotime, China) and then clarified by pre-incubation with the pre-immunized serum, before being subjected to immunoprecipitation by incubation with specific antibodies against Nrf1 or Nrf1D at 4 °C overnight, along with Protein-G Sepharose 4 Fast Flow beads. The immunocomplexes-associated Protein-G Sepharose beads were washed 3 times with PBS. The resulting immunocomplexes with Nrf1 or Nrf1D antibodies were visualized by immunoblotting with the indicated antibodies. For immunohistochemistry analysis, distinct tissues were incubated with the primary antibodies against Nrf1 or Nrf1D and the secondary anti-IgG (Sangon Biotech, Shanghai, China), according to the instructions of manufacturer. The ‘negative’ images were obtained from pre-incubation of pre-immunize serum instead of the primary antibody in the immunohistochemistry.

### 4.9. Subcellular Fractionation

About COS-1 or HepG2 cells (3 × 10^5^) were seeded in 6-well plates and allowed for growth for 24 h in DMEM containing 10% FBS. After reaching the 70% confluence, the cells were transfected with an expression construct for Nrf1 and Nrf1D, or empty pcDNA3.1 vector. Following 24-h recovery from transfection, the cells were harvested and then subjected to incubation with ice cold Nuclei EZ lysis buffer (0.5 mL added to each dish). The nuclei-containing pellets were collected by centrifuging at 500× *g* for 5 min at 4 °C. The supernatant were considered as the non-nuclear fractions, while the sediment nuclear fractions were saved. Subsequently, the sediment was re-suspended by adding 0.5 mL of the nuclei EZ lysis buffer (From Sigma-Aldrich, Saint Louis, MO, USA) and vortexing at moderate to high speeds. After being washed with the lysis buffer for 3 times, the clarified nuclei pellets were collected by centrifuging as described above. The resulting nuclear and non-nuclear fractions were diluted with RIPA lysis buffer (Beyotime, Shanghai, China), and determined by western blotting with distinct antibodies against Nrf1, Nrf1D-specific peptides or their C-terminal V5 tag.

### 4.10. Statistical Analysis

The significant differences in changes of Nrf1- or Nrf1D-mediated reporter gene activity, and the abundances of their transcript and protein expressed in different tissues were determined using the Student’s *t*-test or Multiple Analysis of Variations (MANOVA). The data are herein shown as a fold change (mean ± S.D.), each of which represents at least 3 independent experiments undertaken on separate occasions that were each performed in triplicate (*n* = 9).

## 5. Conclusions

In the present study, Nrf1D is identified as the first candidate secretory transcription factor in mouse blood plasma, although it is largely unknown regarding the detailed mechanisms whereby its precursor, acting as a transmembrane-bound CNC-bZIP protein, is proteolytically processed in close proximity to the ER and unleashed into the blood. Regardless, this disruptive discovery opens a whole new avenue for life scientists to elucidate the gene regulation by Nrf1D secreted possibly from cell communication (even from blood transfusion). Meantime, this exciting work raises a lot of new questions to attract wide attentions from workers in all relevant fields so as to be addressed in the future studies. In addition, we should notice that an inhibitor of DNA binding 1 (ID1) protein was determined as a basic helix-loop-helix (bHLH)-ZIP transcription factor and also allowed to be secreted only in the synovial fluid, but not the blood plasma, of model animals with rheumatoid arthritis [38,39]. However, the small bHLH-ZIP transcription factor does not possess any of the putative transmembrane domains, similar to those within Nrf1D.

## Supplementary Materials

The following are available online at http://www.mdpi.com/1422-0067/19/10/2940/s1.
