# Supplementary material for: Nrf1D Is the First Candidate Secretory Transcription Factor in the Blood Plasma, Its Precursor Existing as a Unique Redox-Sensitive Transmembrane CNC-bZIP Protein in Hemopoietic and Somatic Tissues
Source: Int J Mol Sci. 2018 Sep 27;19(10):2940. doi: 10.3390/ijms19102940 (PMC6213093; doi:10.3390/ijms19102940)
Supplement: Supplementary file 1 [file ijms-19-02940-s001.pdf]

International Journal of
Molecular Sciences

Supplemental Information

Article

Factor in the Blood Plasma, Its Precursor Existing as a Unique Redox-Sensitive Transmembrane CNC-bZIP Protein in Hemopoietic and Somatic Tissues

Jianxin Yuan †, Hongxia Wang †, Yuancai Xiang †, Shaofan Hu †, Shaojun Li, Meng Wang, Lu Qiu and Yiguo Zhang *

Laboratory of Cell Biochemistry and Topogenetic Regulation, College of Bioengineering and Faculty of Sciences, Chongqing University, No. 174 Shazheng Street, Shapingba District, Chongqing 400044, China
* Correspondence: yiguozhang@cqu.edu.cn or eaglezhang64@gmail.com; Tel.: +86-23-6510-2507
† These authors contributed equally to this work.

Figure S1

Idendification of Nrf1D by sequencing of its cDNAs expressed in different tissues

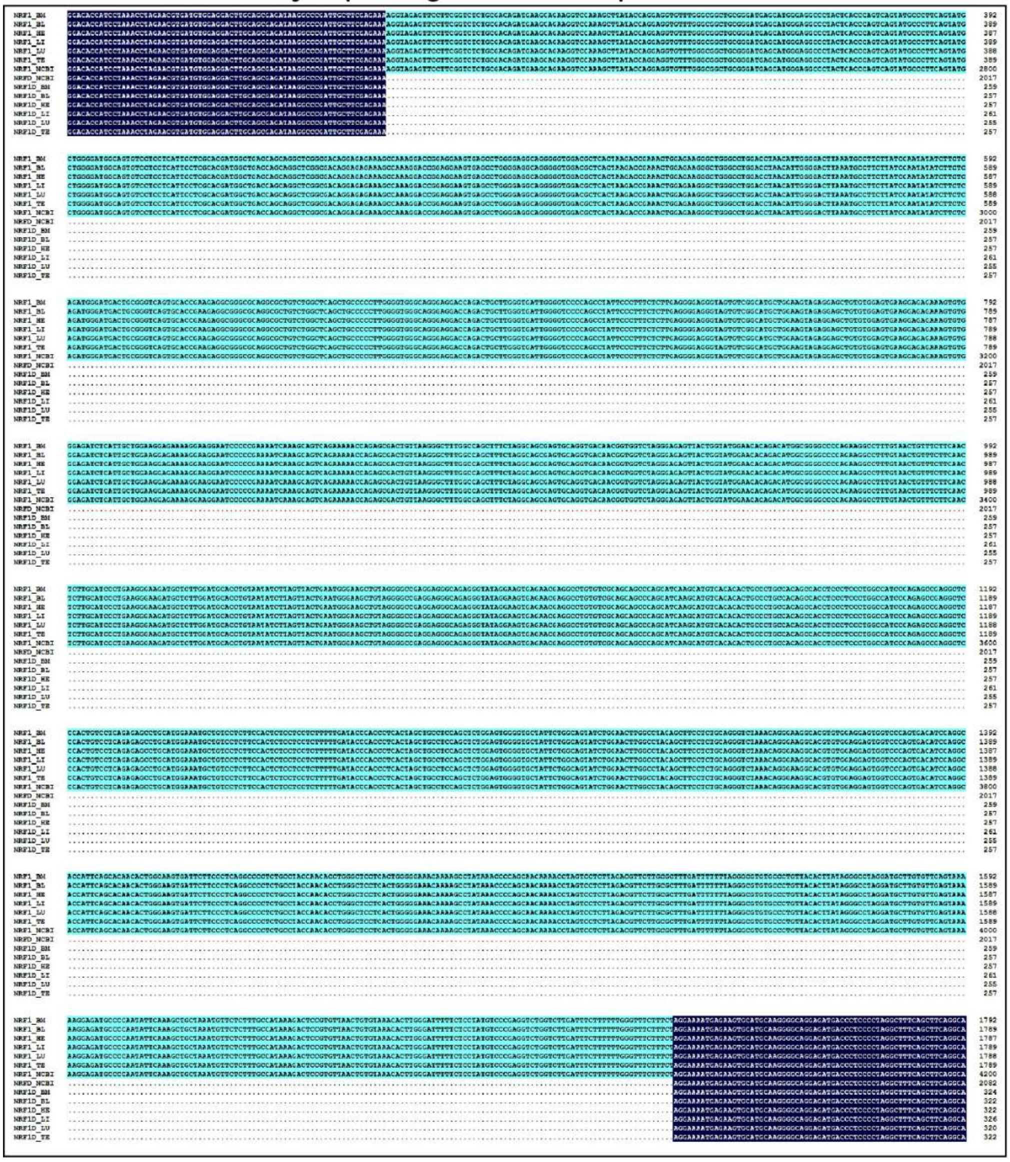

Figure S1. Different transcripts of Nrf1D and Nrf1 expressed in different tissues. The cDNAs from different tissues were produced from RT-PCR (as shown in Figure 1E), and then validated by sequencing. The resultant nucleotide sequences were aligned with both Nrf1D and Nrf1 cDNAs deposited in the GenBank at NCBI. Abbreviations: *BM*, bone marrow; *BL*, blood; *HE*, heart; *LI*, liver; *LU*, lung; and *TE*, testis.

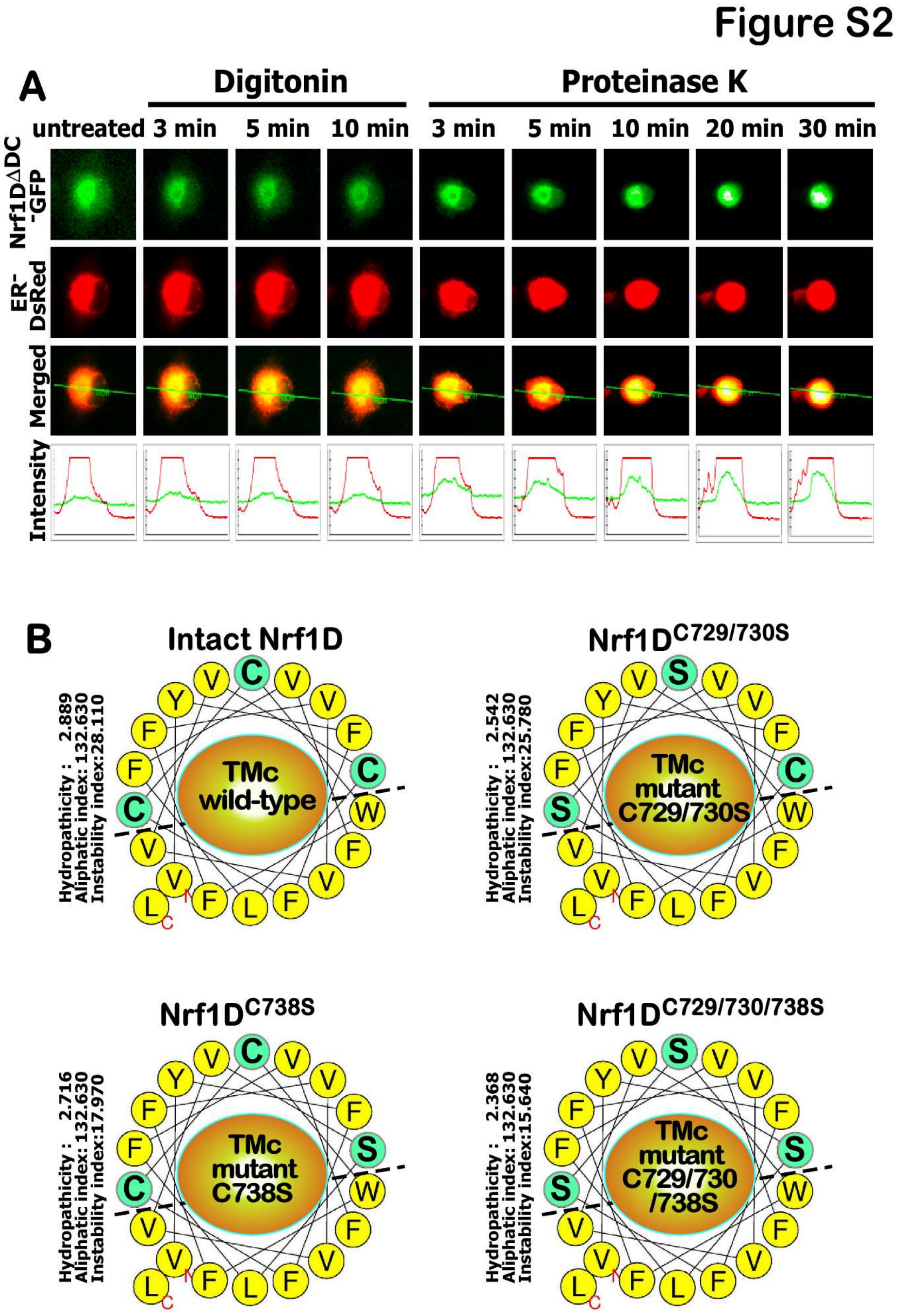

Figure S2. Live-cell imaging of the GFP-fused Nrf1D$^{\Delta DC}$ protein lacking the C-terminal 80-aa region of Nrf1D. (**A**) Live-cell imaging of the Nrf1D$^{\Delta DC}$-GFP fusion protein was performed as described in Figure 5A. For detailed descriptions, see the relevant text of "Materials and methods". (**B**) Four α-helical wheels were made of those amino acids comprising the TMc peptide of Nrf1D, its mutants Nrf1D$^{C729/730S}$, Nrf1D^{C738S} and Nrf1D$^{C729/730/738S}$, respectively. These TMc physico-chemical parameters, including its hydropathicity, aliphatic an instability indexes, were calculated by the ProtParam tool (at https://web.expasy.org/protparam/).

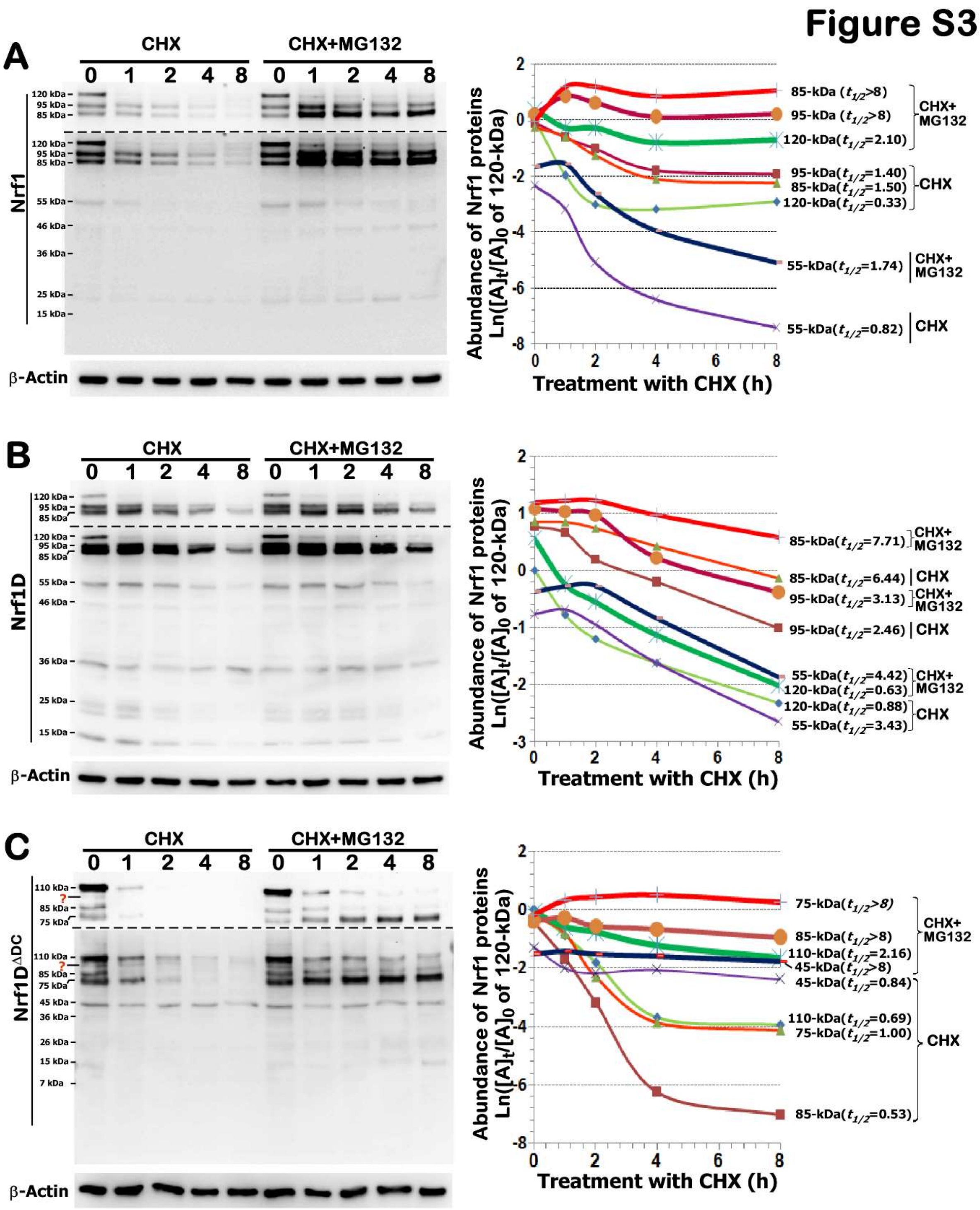

Figure S3. Distinct stability of multiple isoforms derived from Nrf1D in a time-dependent conversion manner. COS-1 cells that had been transfected with an expression construct for Nrf1 (**A**), Nrf1D (**B**) or Nrf1D$^{\Delta DC}$(**C**) and then treated with cycloheximide (CHX, 50 μg/ml) alone or plus 5 μmol/L MG132 for different lengths of time before being disrupted. The total lysates were resolved by 4-12% LDS-NuPAGE gels and then visualized by Western blotting with V5 antibody. The upper images were cropped from the lower pictures, both of which were exposed to development reagents for a longer time. The relative abundances of major isoforms (i.e. ~120-, 95-, 85- and 55-kDa derived from Nrf1, Nrf1D or Nrf1D$^{\Delta DC}$) were quantified by using the Quantity-one software, and their half-lives were also calculated and shown graphically (*right panels*). β-actin served as a protein-loading control.

Figure S4

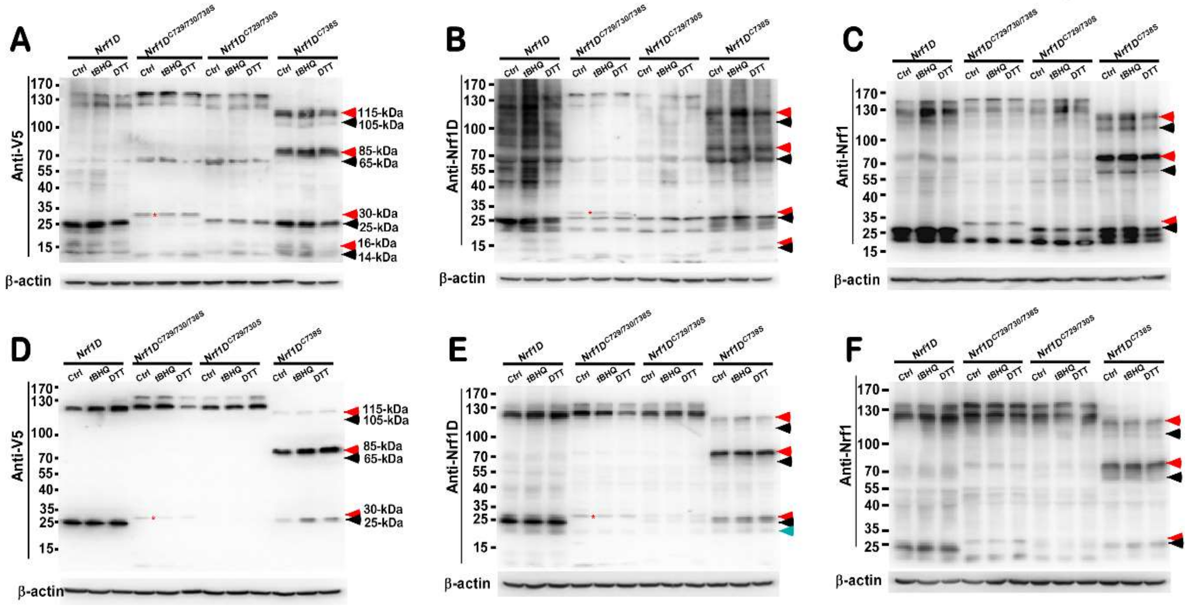

Figure S4. No or less changes in the intracellular abundance of Nrf1D and its TMc mutants in response to redox stress. Both lines of COS-1 (**A**–**C**) and HepG2 (**D**–**F**) cells that had been transfected with an expression construct for Nrf1D, its mutants Nrf1D$^{C729/730S}$, Nrf1D^{C738S} or Nrf1D$^{C729/730/738S}$ were treated with tBHQ (50 μmol/L), DTT (1 mmol/L) for 24 h before being harvested. The total lysates were resolved by SDS-PAGE gels (with upper and lower two-layers containing 8% and 12% polyacrylamide, respectively) and then examined by Western blotting with antibodies against Nrf1, Nrf1D and its C-terminal V5 tag. Some interesting protein bands were indicated by arrows (←) or asterisk (*). β-actin served as a protein-loading control.

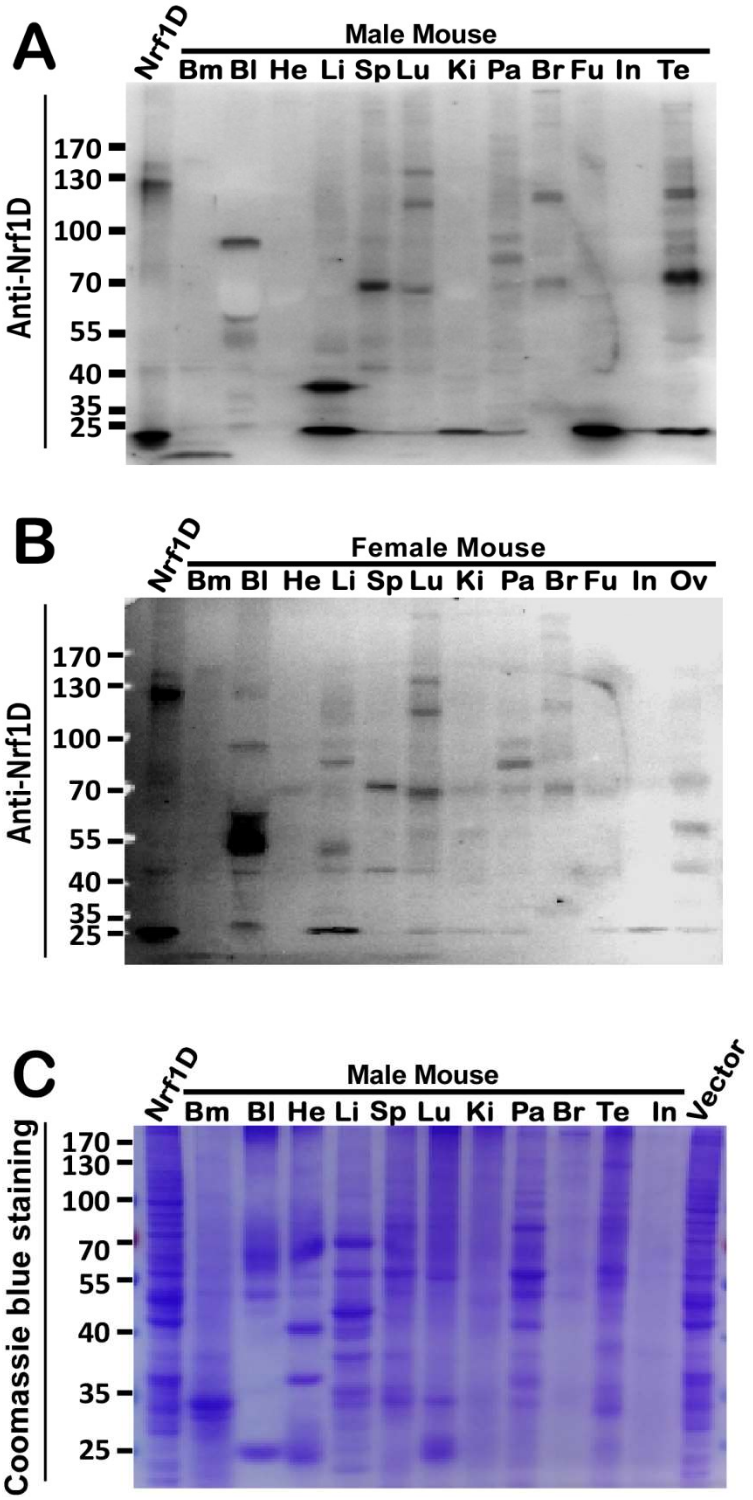

Figure S5. Differential expression of Nrf1D proteins in distinct tissues from male and female mice. Equal amounts of proteins in total lysates extracted from 13 different tissues of male (**A**) and female (**B**) mice were isolated by SDS-PAGE gels and then visualized by Western blotting with Nrf1D-specific peptide antibody. The protein-loaded levels were also seen by Coomassie Blue staining (**C**). Abbreviations: Bm, bone marrow; Bl, blood; He, heart; Li, liver; Sp, spleen; Lu, lung; Ki, kidney; Pa, pancreas; Br, brain; Te, testis; In, intestine; Ov, ovary.
